# How Effective Is Road Mitigation at Reducing Road-Kill? A Meta-Analysis

**DOI:** 10.1371/journal.pone.0166941

**Published:** 2016-11-21

**Authors:** Trina Rytwinski, Kylie Soanes, Jochen A. G. Jaeger, Lenore Fahrig, C. Scott Findlay, Jeff Houlahan, Rodney van der Ree, Edgar A van der Grift

**Affiliations:** 1 Geomatics and Landscape Ecology Research Laboratory, Department of Biology, Carleton University, Ontario, Canada; 2 Australian Research Centre for Urban Ecology, Royal Botanic Gardens Victoria, C/- School of BioSciences, University of Melbourne, 3010, Victoria, Australia; 3 Department of Geography, Planning and Environment, Concordia University Montreal, 1455 Montreal, Quebec, Canada; 4 Institute of the Environment & Ottawa-Carleton Institute of Biology, Ottawa, Ontario, Canada; 5 Department of Biology, University of New Brunswick at Saint John, Saint John, 550, New Brunswick, Canada; 6 Alterra, Wageningen University and Research Centre, Wageningen, The Netherlands; Beihang University, CHINA

## Abstract

Road traffic kills hundreds of millions of animals every year, posing a critical threat to the populations of many species. To address this problem there are more than forty types of road mitigation measures available that aim to reduce wildlife mortality on roads (road-kill). For road planners, deciding on what mitigation method to use has been problematic because there is little good information about the relative effectiveness of these measures in reducing road-kill, and the costs of these measures vary greatly. We conducted a meta-analysis using data from 50 studies that quantified the relationship between road-kill and a mitigation measure designed to reduce road-kill. Overall, mitigation measures reduce road-kill by 40% compared to controls. Fences, with or without crossing structures, reduce road-kill by 54%. We found no detectable effect on road-kill of crossing structures without fencing. We found that comparatively expensive mitigation measures reduce large mammal road-kill much more than inexpensive measures. For example, the combination of fencing and crossing structures led to an 83% reduction in road-kill of large mammals, compared to a 57% reduction for animal detection systems, and only a 1% for wildlife reflectors. We suggest that inexpensive measures such as reflectors should not be used until and unless their effectiveness is tested using a high-quality experimental approach. Our meta-analysis also highlights the fact that there are insufficient data to answer many of the most pressing questions that road planners ask about the effectiveness of road mitigation measures, such as whether other less common mitigation measures (e.g., measures to reduce traffic volume and/or speed) reduce road mortality, or to what extent the attributes of crossing structures and fences influence their effectiveness. To improve evaluations of mitigation effectiveness, studies should incorporate data collection before the mitigation is applied, and we recommend a minimum study duration of four years for Before-After, and a minimum of either four years or four sites for Before-After-Control-Impact designs.

## Introduction

Road traffic kills hundreds of millions of animals every year (reviewed in Seiler [1]), posing a significant threat to many species (e.g., [2–6]). Over forty types of road mitigation measures intended to reduce road-related wildlife mortality (hereafter road-kill) have been implemented or described (reviewed in Hedlund *et al*. [7], Knapp *et al*. [8], Huijser *et al*. [9], Glista *et al*. [10], and van der Ree *et al*. [11]), including those intended to influence motorist behaviour and those intended to influence animal behaviour. The former includes various types of wildlife warning signs, animal detection systems, measures to reduce traffic volume and/or speed, and temporary road closures [9]. The latter includes measures that: scare animals away from the road and/or alert them to approaching traffic; increase the attractiveness of areas away from the road; decrease the attractiveness of the road; and introduce a physical barrier along the road such as fencing with or without safe road crossing opportunities [9]. Many measures are designed to both reduce road-kill and allow wildlife movement across roads, including wildlife warning signs, crosswalks, animal detection systems or crossing structures (under- or overpasses). Moreover, wildlife jump-outs or escape ramps are sometimes integrated with fencing to allow animals to escape from the road corridor should they happen to end up between the fences. Some mitigation measures target specific animal groups. For example, measures targeting large mammals, often ungulates in particular, include wildlife reflectors and mirrors, animal detection systems, and roadway lighting. Fencing has also been designed to take into consideration the climbing or burrowing ability of animals. For example, fences can be modified with top extensions (e.g., a ‘floppy top’ or ‘overhanging lip’), or built with a smooth vertical surface, to prevent animals from climbing over them, or the base of the fence can be buried or include a skirt to prevent animals from digging under and breaching the fence [12].

Considering the variety of mitigation measures currently available to reduce road-kill, deciding on what method to implement has been a contentious issue. This issue largely stems from two considerations: (1) costs of mitigation can be extremely variable, and (2) there is little reliable information about the relative effectiveness of these measures in reducing road-kill. Economic considerations strongly influence the chosen mitigation measure [10]. Comparatively inexpensive measures (e.g. warning signs, wildlife reflectors, whistles or repellents) are commonly employed by transportation agencies despite there being little evidence concerning their effectiveness [7–9, 13]. For example, wildlife warning signs are perhaps the most common mitigation measure implemented in the United States to reduce large animal collisions with vehicles, yet many state transportation and natural resource agencies reported they did not know whether this measure was effective [14, 15]. In contrast, measures that are thought to be more effective (i.e., wildlife fencing, crossing structures, and animal detection systems for large mammals) may not be implemented due to high cost and low public support [9, 16]. Where cost, rather than effectiveness, drives decision-making, mitigation effectiveness may be compromised [10].

Among the more expensive mitigation measures, the question remains as to whether combining fences with crossing structures is more effective than fences or crossing structures alone. It is commonly suggested that to reduce road-kill, wildlife crossing structures (over- and under-passes) should always be combined with wildlife fencing (e.g., [9, 17–19]). This recommendation stems from the understanding that the primary function of wildlife fencing is to keep animals off roadways, whereas the primary function of wildlife crossing structures is to provide safe crossing opportunities so as to reduce the barrier effect of the roads and/or associated fencing. However, wildlife crossing structures may be installed with little or no associated fencing, particularly for species where fencing is not feasible or considered too expensive. Furthermore, animals may be more likely to break through wildlife fencing if safe crossing opportunities are not provided, prompting further recommendations to implement crossing structures in combination with wildlife fencing [9]. These considerations suggest that the effectiveness of crossing structures with associated fencing in reducing road-kill should be greater than the effectiveness of fencing or crossing structures alone.

Adding to the challenge for decision-makers is that information on mitigation effectiveness is often based on studies that permit, at best, weak inference, and yield low predictive power [20]. Studies evaluating mitigation effectiveness often lack: (1) comparisons between impact sites (i.e., sites where mitigation measures are installed or modified) and control sites (i.e., sites where a road is present but there is no mitigation or modification); (2) data collection before the mitigation is applied; (3) replication in space and time; and (4) randomization of impact and control sites across the pool of potential study sites (see Roedenbeck *et al*. [20], van der Grift *et al*. [21], Rytwinski *et al*. [22], and van der Ree *et al*.[23], for further details on how to improve road mitigation research). The paucity of good-quality studies on mitigation effectiveness has made it difficult for transportation agencies to make informed decisions as to which method to use. Furthermore, while there are many studies of effectiveness of mitigation measures (e.g., [24–27]), these studies address individual cases, in particular sites and on particular species. To date, reviews of these studies do not combine them to generate statistically defensible conclusions across mitigation types and/or taxa (e.g., [9, 10]).

Here, we present the first comprehensive analytical review of the effectiveness of road mitigation measures in reducing road-kill using well-described meta-analysis methods. Unlike qualitative syntheses (e.g., systematic reviews) such an approach permits quantitative estimates of the overall effectiveness (i.e. effect size) of different mitigation measures, identify factors associated with variation in effect sizes among studies, and provide directions for future research by identifying issues or questions which cannot be resolved/answered with currently available data.

The purpose of this study was to employ standard meta-analytic methods to ask: (1) To what extent does road-kill mitigation effectiveness differ among measures? For example, are fences with crossing structures more effective than fences or crossing structures alone? Are less expensive measures such as reflectors as effective as fencing and/or crossing structures?; (2) To what extent do taxa differ in the effectiveness of particular road mitigation measures?, and (3) To what extent does study design influence the estimated effectiveness of road mitigation measures?

## Materials and Methods

### Search strategy and study selection

We searched for studies (journal articles, reports, conference proceedings, theses) that quantified the relationship between road-kill and a mitigation measure that was installed, at least in part, to reduce wildlife road mortality. Our indices of road-kill included: (1) dead animal counts determined either by carcass removal data (collected by road maintenance personnel or by employees of natural resource management agencies) or by carcass observations (collected by researchers or the public), or (2) the number of reported wildlife-vehicle collisions. Literature searches were conducted in ProQuest Dissertations and Theses database (Sept 2014), ProQuest Science, Technology & Medicine database (Oct 2014), and ISI Web of Science database (Nov 2014), using the following keyword string: (“road*”, “highway*”, OR “traffic*”) AND (“wildlife*”, “fauna*”, “animal*”, “amphibian*”, “reptile*”, “mammal*”, “ungulate*”, “bird*”, “invertebrate*”, “insect*”, OR “butterfly*”) AND (“culvert*”, “tunnel*”, “passage*”, “overpass*”, “underpass*”, “bridge*”, “pole*”, “fenc*”, “crossing structure*” OR “mitigation*”). No particular date, document type, country, or language constraints were applied. We also searched Google Scholar (100 first hits Nov 2014) using combinations of the keywords included in the above search string. Only English language search terms were used. In addition, we searched specialist conservation and government websites for data and for relevant experts, practitioners, and consultants who were subsequently invited to identify candidate studies. We also searched papers and abstracts published in the conference proceedings of the International Conference on Wildlife Ecology and Transportation (ICOWET)/ International Conference on Ecology and Transportation (ICOET) and the Infra Eco Network Europe (IENE). In addition, reference lists from a number of relevant reviews and reports (e.g., [9, 10, 28, 29]), and road ecology books (e.g., [11, 30, 31]) were examined, as were the lists of references of all sources we reviewed. We also conducted targeted searches of a number of road ecology research center websites: Road Ecology Center, University of California at Davis; Center for Transportation and the Environment, North Carolina State University; Western Transportation Institute, Montana State University; and Brazilian Center of Studies in Road Ecology (Centro Brasileiro de Estudos em Ecologia de Estradas).

Only primary empirical studies were included i.e., we excluded data from review papers, anecdotal reports, and simulation studies. We limited our analyses to include only animals (vertebrates and invertebrates) that are terrestrial for at least part of their life cycle. Any mitigation measure intended to reduce road-kill was included: animal detection systems, wildlife warning signs, changes in road-verge management, measures to reduce traffic volume and speed, temporary road closures, wildlife crossing structures (e.g., under- or over-passes: amphibian tunnels, badger pipes, ledges in culverts, land bridges, rope bridges, glider poles), wildlife fences, wildlife mirrors, wildlife reflectors, wildlife chemical repellants, population reductions (e.g., culling), wildlife whistles, and modified road designs/viaducts/bridges/lighting. We also included wildlife crossing structures—a measure primarily intended to increase wildlife movement across roads—in situations where at least one of the goals was to reduce road-kill, as indicated by the fact that authors measured road-kill.

Studies included in the analysis employed one of three study designs: Control-Impact (CI), Before-After (BA), and Before-After-Control-Impact (BACI). CI studies provided comparisons of road-kill at impact and control sites. 'Impact' sites were locations where a mitigation measure was installed and/or modified, and 'control' sites were locations where a road was present but there was no mitigation, or modification. In BA designs, road-kill was measured and compared before and after the mitigation measure was installed or modified. In BACI designs, road-kill was measured before and after the mitigation measure was installed or modified, both at sites with the installation/modification and at control sites without the installation/modification.

Studies that reported means and sample sizes but no associated variances were excluded from the review (*n* = 5). To be included, studies had to have a total sample size of≥ 4, as this is necessary for calculating an effect size (see Raw effect size calculations below).

To reduce possible impacts of publication bias and ensure comprehensive coverage, we included studies published in any print outlet, including peer-reviewed scientific journals, government reports (e.g., state department of transportation reports), conference proceedings, consultant reports and theses. Because the same data may be reported in several publications, we screened all studies for duplicate datasets and used data from the most complete source.

### Data extraction

From each study, we extracted sample sizes, means, and associated variances for both impact and control sites, and/or before and after mitigation installation/modification, and/or a test statistic that could be converted into an effect size. In cases where these summary statistics were not explicitly provided, we calculated them using raw data if these data were published (e.g., in an appendix), could be extracted from graphical images using GetData Graph Digitizer 2.26 (Fedorov S. (2013), unpublished internet freeware), or were provided to us by the authors.

Many papers reported the effects of mitigation measures on several species or taxa. In such cases, we calculated multiple effect sizes, one for each species/taxon. Some studies included results from what were, in effect, different studies (e.g., a comparison of road-kill at multiple sites before and after mitigation in two different study locations, each location involving a different set of roads) in which case studies were considered independent.

We did not attempt to evaluate the quality of the road-kill data. We used the number of years monitored (before and after) as sample size for BA studies, irrespective of the sampling effort, and the number of sites (impact and control) as sample size for CI studies. The determination of sample sizes for BACI designs is described below.

### Adjustments prior to effect size calculations

To control for potential differences in sampling effort among studies, we divided road-kill counts at impact and control sites by the length of road surveyed in km if the reported measure did not already do so. When road mitigation measures were implemented during the study period, we removed observations taken during the construction phase if they could be identified. Where not possible, we calculated an effect size based on all the data provided and noted our inability to distinguish the “during construction” phase. Moreover, if there were multiple phases of road and/or mitigation construction/modification, we considered the “after” phase to begin only when all construction/modification had been completed.

For CI and BA studies, we calculated means and standard deviations across sites or over years. If there was only a single site (CI studies) or a single year (BA) of data in any class (i.e. before or after; control or impact) we treated this value as a mean and set the standard deviation the same as the other class (*n* = 12 studies).

For BACI studies, the interaction effect between treatment and year is usually the effect of interest to the researchers and is often reported. However, it is often difficult to calculate an effect size from the interaction effect, unless the raw data are also presented in the study. To get an effect size estimate for BACI studies we either aggregated data over years and compared over sites or aggregated over sites and compared over years. We aggregated (1) based on how the data were reported, and/or (2) to maximize sample size. If the data were compared over sites, we used the number of sites as the sample size, and if we compared over years, we used the number of years as the sample size.

### Raw effect size calculations

We used the standardized mean difference (Hedges’ *d*) as our effect size measure [32, 33]:

$$d=\frac{{\bar{X}}_{G1}-{\bar{X}}_{G2}}{s_{pooled}}J$$ (1)

where ${\bar{X}}_{G1}$ and ${\bar{X}}_{G2}$ are the means of group 1 (G1 = either control sites or before monitoring period) and group 2 (G2 = either impact sites or after monitoring period), *s*_pooled_ is the pooled standard deviation of the two groups, and *J* is a correction term that removes small sample size bias [34],

$$J=\left[1-\frac{3}{4N-9}\right],$$ (2)

where *N* = total sample size.

Thus, the effect size *d* is the difference in standard deviation units between the means of group 1 and group 2. A positive *d* indicates a reduction in road-kill with the road mitigation and a negative *d* indicates an increase in road-kill with the road mitigation. The sampling variance of is given by:

$$se=\sqrt{\frac{n_{G1}+n_{G2}}{n_{G1}n_{G2}}+\frac{d^{2}}{2\left(n_{G1}+n_{G2}\right)}}$$ (3)

### Data analysis

We used two different but related datasets in our analyses. The complete dataset (*n* = 99) treated each effect size estimate as independent. By contrast, the synthetic effect size dataset (*n* = 67) was derived by pooling multiple effect sizes corresponding to different taxa from a single study. Pooling multiple effect sizes within a single study reduces the effective sample size and decreases the weight of correlated and extreme estimates of effect size, thereby leading to more statistically conservative results [35, 36]. As results were qualitatively similar using both datasets, we here report only the complete dataset analysis (see S1 Text for statistical methods and results for the synthetic effect size dataset).

To determine whether mitigation measures reduce road-kill, we first conducted a random-effects meta-analysis using the DerSimonian-Laird method [37, 38] to determine the summary weighted-mean effect size using the complete dataset (*n* = 99). In contrast to a fixed-effects model, a random-effects model assumes that the true effect size will vary from study to study and that there is no single common underlying effect size. Thus, the conclusions of a random-effects model are typically generalizable to a larger, unknown group of similar studies. Under a random-effects model, the weight assigned (w*) to each effect size is the inverse of the sum of two variance components w* = 1/ (*w* + *τ*^*2*^), where *w* (= 1/ se^2^) is the unique sampling variance for each study (within-study error) and *τ*^*2*^ is the pooled variance of the true effects across all studies (between-studies variance). We also calculated the heterogeneity in true effects (*Q* statistic), which we compared against a chi-square distribution, to test whether the total variation in observed effect sizes (*Q*_*T*_) was significantly greater than that expected from sampling error (*Q*_*E*_).

To address our research questions (summarized in S1 Table), we investigated a set of candidate predictor variables (Table 1) from four broad categories: attributes of (a) planning and management; (b) wildlife; (c) fencing; and (d) study design. In most cases, we collected predictor variable information from the same source as the extracted effect size. In a few instances, we retrieved relevant information from other sources by the same author.

**Table 1:** Candidate predictor variables by category.

Predictor category	Predictor variable	Description
*Planning/Management*		
	Road type	road category where the road mitigation measure was studied: ≥4 lane divided highway versus 1-2-lane roads
	Mitigation category	Crossing structure + Fencing; fencing only; crossing structure only; animal detection systems; wildlife reflectors; other mitigation measures
*Wildlife*		
	Taxon	birds; combination of amphibians and reptiles; large mammals ≥10kg; small to medium sized mammals <10kg
*Fencing*		
	Type	large mammal fence; small-medium sized mammal fence; amphibian and reptile fencing
	Length	Average length of fencing (m)
*Study design*		
	Study design	BA = before-after, BACI = before-after-control-impact, CI = control-impact study designs
	Total study duration	# of before years + # of after years
	Were data collected during construction of mitigation excluded?	Were mortality data collected during construction of the mitigation excluded? YES/NO
	Was mortality beyond fence-ends included in data?	Was wildlife mortality monitored at a certain distance beyond the ends of the fencing and included in the effect size estimate to control for the potential issue of increased mortality at fence-ends? YES/NO

We used mixed-effects meta-regression to examine associations between effect size and candidate predictor variables using restricted maximum-likelihood to estimate heterogeneity [38–40]. Meta-regression analysis was conducted in R 3.0.3 [41], using the ‘metafor’ package (version 1.9–4) [42]. We adopted two approaches depending on whether or not we had an *a priori* hypothesis for the candidate predictor in question (summarized in S1 Table). Where we had no *a priori* hypothesis, we evaluated fitted models using Akaike Information Criterion (AICc) and *R*^*2*^, and accompanied by corresponding *Q*_*E*_ (test statistic of residual heterogeneity) and *Q*_*M*_ (Omnibus test statistic of covariates). We determined there was an association between effect size and candidate predictor variable(s) if the mixed-effects model had a lower AICc than the null model i.e., random-effects model with no predictor. For candidate predictors for which we had *a priori* hypotheses, we used the subset of effect sizes appropriate for testing the hypothesis in question. For example, to answer the question of whether there is an additional benefit (in terms of reducing road-kill) to fencing associated with crossing structures, the appropriate comparison involved studies with crossing structures only versus crossing structures with associated fencing. We then evaluated fitted models using *p*-values (one-tailed, *p*<0.05 significance level) and confidence intervals.

We used the coefficient of determination (*R*^2^) from the meta-regression models to estimate the predictive value of candidate predictor variables, or sets of variables. We assessed heterogeneity using: weighted sum of squares (*Q*); tau-squared (_*T*_^*2*^), an estimate of between-studies variance; the proportion of observed variance that reflects real differences in effect size (*I*^*2*^); and the ratio of total variability to sampling variability (*H*^*2*^). Given the comparatively small number of effect sizes (total *n* = 99), we restricted the number of fitted parameters (*k*) in any candidate model such that the *n/k* ratio was greater than 5, sufficient in principle to ensure reasonable model stability and sufficient precision of coefficients [43].

Since information on mitigation measure attributes were not always provided in studies, when investigating associations between attributes and effect sizes, we attempted to maximize the number of effect sizes with complete information on as many attributes as possible by removing effect sizes with missing information. Because the sample size for amphibians was small (*n* = 4), we combined amphibians and reptiles for all analyses. Mammals were categorized into two size classes: (1) small to medium sized mammals (< 10 kg), and (2) large mammals (≥ 10 kg). Some continuous predictor variables were log-transformed to meet test assumptions.

We tested for publication bias using funnel plots of asymmetry i.e., graphical detection of publication bias using a scatterplot of effect size vs. sampling error, as well as Egger’s regression test for funnel plot asymmetry [42].

As effect sizes may not be easily interpretable, we attempted to convert *d* to a percent change in mitigation effectiveness by plotting the relationship between *d* and the percent change in mitigation effectiveness:

$$\left[\frac{\left({\bar{X}}_{G1}+q\right)-{\bar{X}}_{G2}}{{\bar{X}}_{G1}+q}\right]*100,$$ (4)

where ${\bar{X}}_{G1}$ and ${\bar{X}}_{G2}$ are the means of group 1 (G_1_ = either control sites or before monitoring period) and group 2 (G_2_ = either impact sites or after monitoring period). Since percent change cannot be computed when ${\bar{X}}_{G1}=0$, we added a small constant *q* = 0.01 to ${\bar{X}}_{G1}$ for each effect size estimate within the dataset.

## Results

### Description of studies

We found 140 studies published from1981 to April 2015 that examined the effectiveness of road mitigation measures in reducing road-kill. Only 50 of these met our inclusion criteria, about half of them (27) from grey literature (refer to S2 Table and S1 Reference List for studies included in the meta-analysis). We excluded studies for the following reasons: (1) data from the same study was reported in multiple publications (~46.5%; 42 studies), (2) total sample size was too small i.e., *n* < 4 (~17%; 11 studies), or (3) insufficient information were provided to calculate an effect size (~36.5%; 33 studies) (S1 Fig).

**Fig 1:**
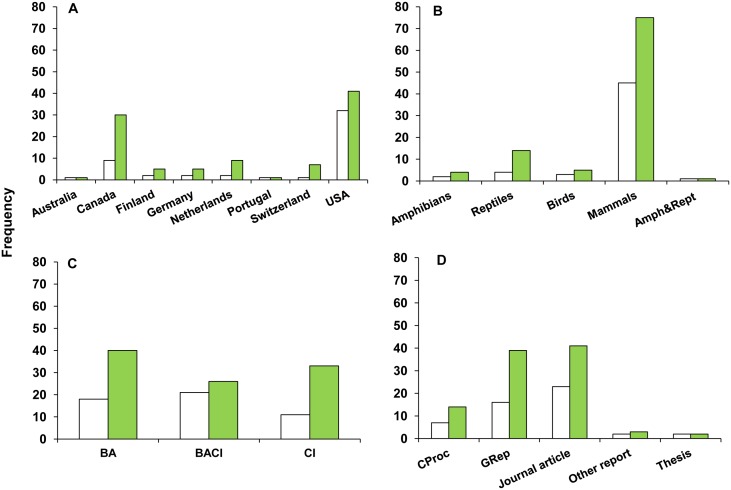
Number of studies (white bars, including conference proceedings (CProc) and government reports (GRep)) and effect size estimates (solid bars) in relation to (A) country, (B) taxon, (C) study design, and (D) publication type. Amph&Rept: effect sizes combined amphibians and reptiles; BA: Before/After; BACI: Before-After-Control-Impact; CI: Control/Impact study designs.

The 50 included studies generated 99 effect size estimates. Studies were predominantly from North America (41), with some from Europe (8), and Oceania (1) (Fig 1A). Forty-five studies from 8 countries included mammals (e.g., *Odocoileus hemionus*, *Ovis Canadensis*, *Procyon lotor*, *Erinaceus europaeus*), yielding 75 effect size estimates (Fig 1B and refer to S2 Table for full list of included taxa). Three studies from 2 countries included birds (e.g., *Anas platyrhynchos*, *Sterna maxima*), yielding 5 effect size estimates. Two studies from 2 countries included amphibians (e.g., *Lithobates sylvaticus*, *Anaxyrus americanus americanus*) resulting in 4 effect size estimates, and 4 studies from 3 countries included reptiles (e.g., *Chrysemys picta*, *Zootoca vivipara*) resulting in 14 effect sizes. Sixty-seven percent of studies used a BACI or BA study design (Fig 1C).

**Fig 2:**
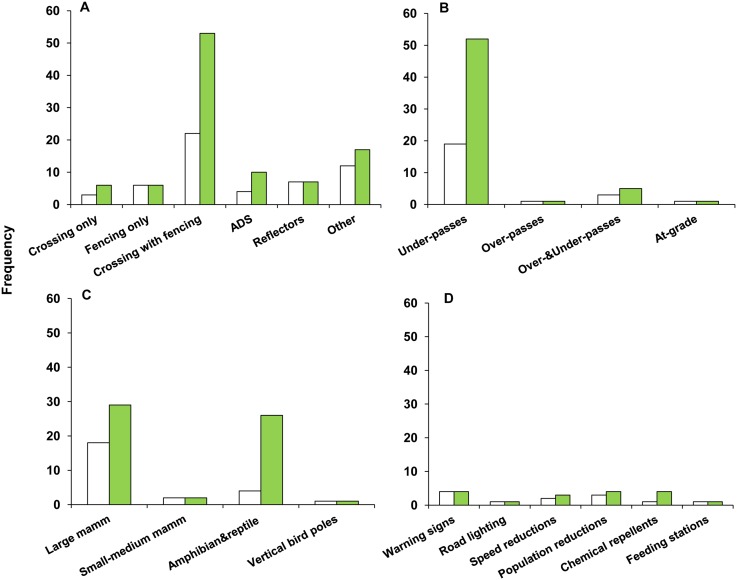
Number of studies (white bars) and effect size estimates (solid bars) in relation to (A) mitigation type, (B) crossing structure type, (C) fencing type, and (D) other mitigation types. Crossing: crossing structures; Crossing with fencing: combination of crossing structures and associated fencing; ADS: animal detection systems; Reflectors: wildlife reflectors; Other: other mitigation types e.g., wildlife warning signs; Mamm: mammal.

Most studies with effect size estimates concerned crossing structures with associated fencing (Fig 2A), the overwhelming majority of which were under-passes (Fig 2B). Fencing studies were mainly on fencing for large mammals (Fig 2C). After crossing structures with fencing, the two most common mitigation measures evaluated were wildlife reflectors and animal detection systems (Fig 2A and 2D).

### Global analysis and publication bias

**Fig 3:**
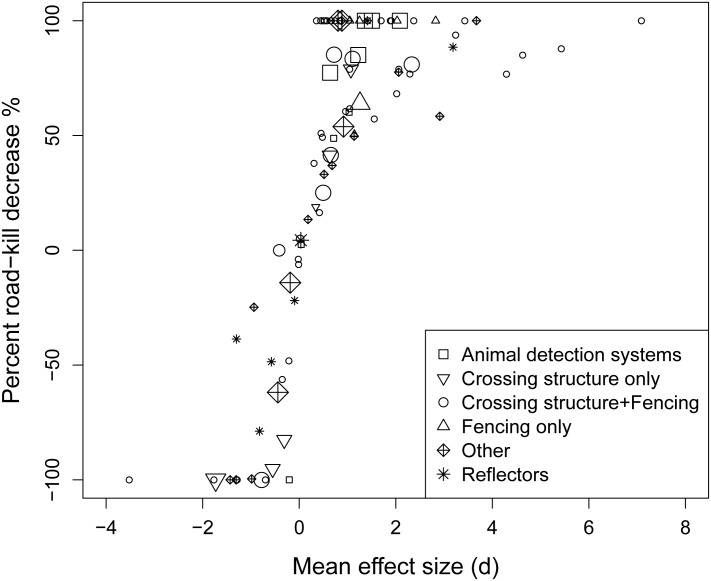
Relationship between mean effect size and the percent road-kill decrease [Eq 4] (*n* = 99 effect sizes). Symbol size is proportional to the weight (inverse of the sampling variance) of the effect size; smaller symbols correspond to effect sizes with lower weights.

The overall mean weighted effect size was 0.75 (95% CI: 0.50, 1.00), corresponding to a roughly 40% overall decrease in road-kill between impact and controls (Fig 3). There was however, substantial heterogeneity in effect sizes (*Q* = 239.44, *p* < 0.0001, *n* = 99; _*T*_^*2*^ = 0.85; *I*^*2*^ = 59.07%; *H*^*2*^ = 2.44), indicating that there was substantial variation in road-kill reduction. Egger’s regression test (*z* = 2.04, *p* = 0.041) suggested possible evidence of publication bias towards studies showing reduced road-kill with implementation of mitigation (S2 Fig). When separating peer-reviewed publications from non-peer-reviewed studies, evidence of publication bias was only present in the former (*z* = 3.74, *p* = 0.0002, *n* = 42, *z* = -1.06, *p* = 0.291, *n* = 57, respectively), suggesting that journals may be more likely to publish studies showing effectiveness of mitigation measures rather than ineffectiveness.

**Fig 4:**
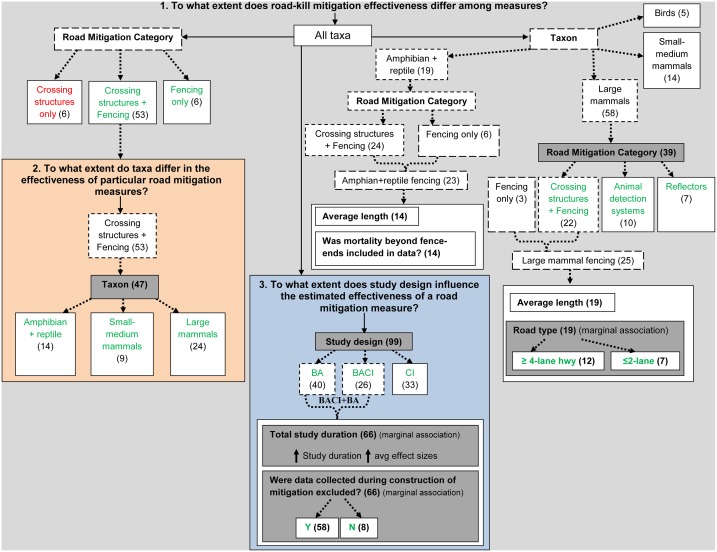
Summary flow chart of the meta-analysis addressing our three main research questions using the complete dataset of effect sizes (*n* = 99) and appropriate subsets (dashed boxes). Boxes enclosed by solid lines indicate predictor variables or subset categories under consideration. Shaded predictors were associated with road mitigation effectiveness. Subset categories in green indicate an overall average reduction in road-kill with road mitigation; red indicates an overall average increase in road-kill with road mitigation. Values in parentheses are the number of effect sizes. BA: Before-After; BACI: Before-After-Control-Impact; CI: Control-Impact study designs. (See S1 Table for a complete list of research questions and predictor variables)

The following sections address our research questions listed in Fig 4 (and in S1 Table).

### 1. To what extent does road-kill mitigation effectiveness differ among measures?

#### Overall, do crossing structures with associated fencing enhance the road-kill reduction effects of fencing per se?

**Fig 5:**
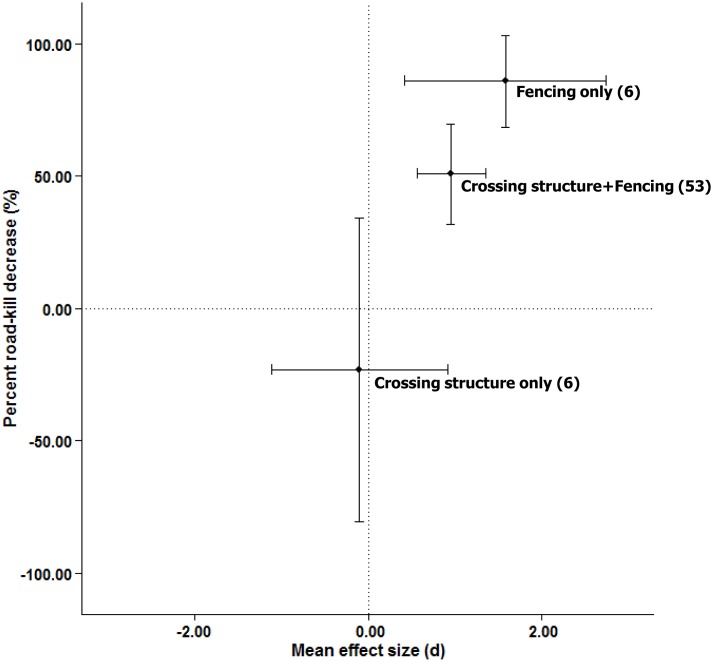
Relationship between weighted-mean effect sizes and the weighted-mean percent road-kill decrease for crossing structures and fencing alone and in combination. Values in parentheses are the number of effect size estimates. Error bars indicate 95% confidence intervals.

The average effect size for crossing structures with associated fencing was no greater than the average effect size for fencing alone (*Q*_*M*_ = 1.00, *p* = 0.841(one-tailed), *R*^*2*^ = 0.34, *n* = 59; 50.7% versus 85.8% reduction in road-kill, respectively), indicating no detectable additional reduction in road-kill afforded by adding crossing structures to fencing (Figs 4 and 5).

#### Overall, does fencing associated with crossing structures enhance the road-kill reduction effects of crossing structures per se?

The average effect size for crossing structures with associated fencing was greater than that of crossing structures alone (*Q*_*M*_ = 3.53, *p* = 0.030 (one-tailed), *R*^*2*^ = 8.97, *n* = 59; 50.7% reduction versus 23.1% increase in road-kill, respectively), indicating there is an additional benefit to adding fencing (Figs 4 and 5).

#### What mitigation measures are most effective for large mammals?

**Fig 6:**
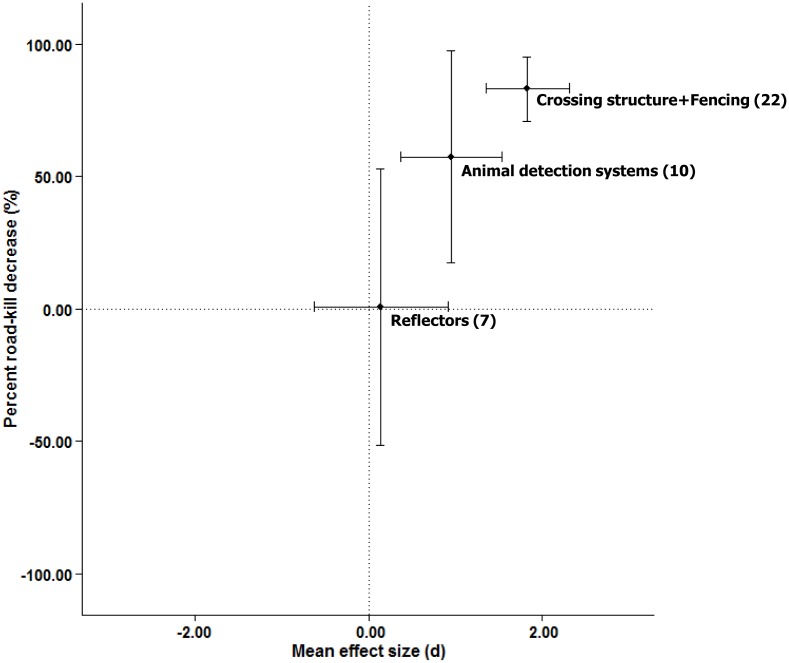
Relationship between weighted-mean effect sizes and the weighted-mean percent road-kill reduction for three different types of mitigation measures, based on a sample of *n* = 39 large mammal effect sizes. Values in parentheses are the number of effect size estimates. Error bars indicate 95% confidence intervals.

Crossing structures with associated fencing and animal detection systems had larger average effect sizes than wildlife reflectors (Table 2A; Figs 4 and 6), but there were too few studies of large mammal fencing without crossing structures to evaluate the effectiveness of large mammal fencing alone.

**Table 2:** Associations between effect sizes and (A) mitigation category (*n* = 39); (B) road type (*n* = 19), for the subset of studies involving large mammal fencing. Mitigation category: Crossing structures with associated fencing, animal detection systems, and wildlife reflectors; Road type: ≥4- lane divided highways, and 1–2 lane roads. *Notes*: Null model = random-effects model.

Predictor	AICc	*R*^2^	*Q*_*E*_	*Q*_*M*_
(A)				
Null model	136.17	-	-	-
Mitigation category	128.16	52.81	61.18 (*p* = 0.006)	14.53 (*p* = 0.001)
(B)				
Null model	69.17	-	-	-
Road type	68.19	26.00	31.43 (*p* = 0.018)	3.99 (*p* = 0.046)

#### What mitigation measures are most effective for small to medium sized mammals, amphibians and reptiles, and birds?

There was insufficient variation among mitigation categories to permit meaningful tests for taxa other than large mammals.

#### Which attributes of the most common measures are associated with effectiveness?

Fencing and crossing structures are often designed with specific taxa in mind. To reduce the potential confounding effect of taxon, fencing and crossing structure attributes were evaluated separately for different taxa.

Fencing. For large mammal fencing, fence length and road type were the only attributes with sufficient sample size and variation to permit meaningful tests. Road type was associated with average effect sizes (Table 2B), with large mammal fencing associated with larger effect sizes along 4 (or more) lane divided highways than along 1–2 lane roads. We found no detectable association between fence length and average effect sizes.

For amphibian and reptile fencing, fence length and whether or not road-kill was monitored beyond the ends of the fencing to control for potential increased mortality at fence-ends were the only attributes for which sample size and variation were sufficient to permit meaningful tests. Neither of these two variables was associated with average effect sizes. Other taxa (e.g. small and medium-sized mammals) could not be investigated owing to inadequate sample sizes.

Crossing structures. There were too few effect sizes with complete information on crossing structure attributes to permit meaningful analysis.

### 2. To what extent do taxa differ in the effectiveness of particular road mitigation measures?

**Fig 7:**
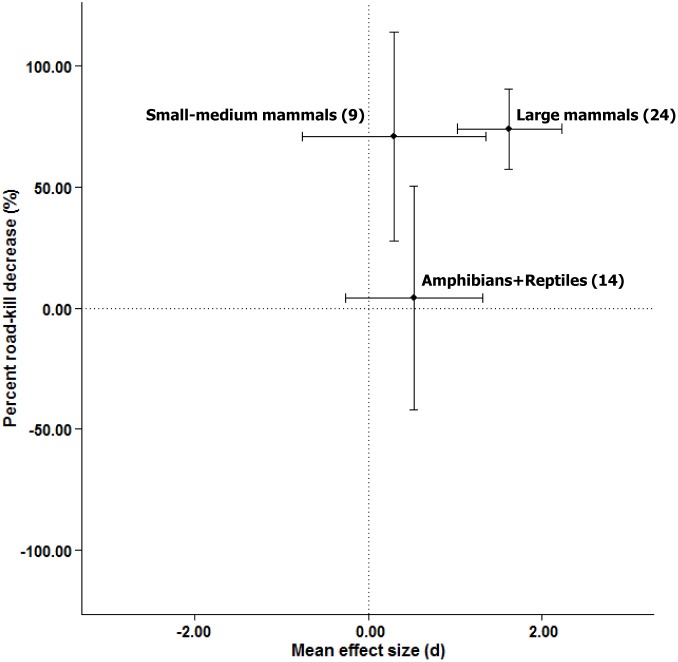
Relationship between weighted-mean effect sizes and the weighted-mean percent road-kill reduction for different taxa, based on *n* = 47 effect sizes from studies involving crossing structure and associated fencing. Values in parentheses are the number of effect size estimates. Error bars indicate 95% confidence intervals.

There was only sufficient sample size within the crossing structures and associated fencing mitigation category to address this question. The effectiveness of crossing structures with associated fencing in reducing road-kill varied among taxa [AICc (null) = 188.20; AICc (Taxon) = 185.84; *R*^*2*^ = 18.73, *n* = 47; *Q*_*E*_ = 117.54 (*p* < 0.0001); *Q*_*M*_ = 7.10 (*p* = 0.029)], with large mammals having larger average effect sizes than small to medium sized mammals and amphibians and reptiles (Figs 4 and 7).

### 3. To what extent does study design influence the estimated effectiveness of a road mitigation measure?

**Fig 8:**
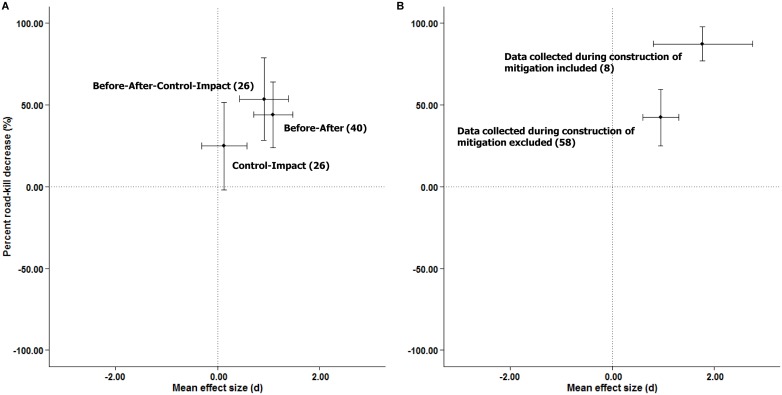
Relationship between weighted-mean effect sizes and the weighted-mean percent road-kill reduction for (A) study design, and (B) whether mortality data collected during construction of the mitigation measure was excluded, based on studies employing BA or BACI designs. Values in parentheses are the number of effect size estimates. Error bars indicate 95% confidence intervals.

BA and BACI designs had larger average effect sizes than CI studies (Figs 4 and 8A; Table 3A). But even within the BA and BACI designs there is considerable heterogeneity in effect size (*Q* = 190.29, *p* < 0.0001, *n* = 66). We found that studies that included mortality data obtained during construction of the mitigation measure had larger average effect sizes than those that did not (Figs 4 and 8B), as did studies of longer duration, though in both cases the association is weak (S3 Fig). A multivariate model including both variables was more informative than either univariate model (Table 3B).

**Table 3:** Study design type predictor variables showing associations with effect sizes for: (A) the complete dataset (*n* = 99), and (B) the combination of Before-After and Before-After-Control-Impact subset (*n* = 66). “During construction data separation” means that mortality data collected during construction of the mitigation was excluded from analyses. *Notes*: Null model = random-effects model.

Moderator(s)	AICc	*R*^*2*^	*Q*_*E*_	*Q*_*M*_
(A)				
Null model	352.77	-	-	-
Study design	346.22	16.06	217.98 (*p* < 0.0001)	11.17 (*p* = 0.004)
(B)				
Null model	243.55	-	-	-
Total study duration	243.09	9.65	176.80 (*p* < 0.0001)	2.68 (*p* = 0.101)
During construction data separation	243.25	9.63	177.73 (*p* < 0.0001)	2.53 (*p* = 0.112)
Total study duration + During construction data separation	243.13	18.40	166.71 (*p* < 0.0001)	5.24 (*p* = 0.073)

## Discussion

### Global analysis and publication bias

Overall, mitigation measures reduce road-kill by approximately 40% compared to controls. This result did not change when considering each effect size as an independent observation or when pooling effect sizes over taxa within studies (S1 Text). The overall heterogeneity of effect sizes was large, indicating that there was considerable variation among estimates in the extent to which mitigation measures reduced road-kill.

In addition to possible evidence of publication bias (S2 Fig), there were some geographical and taxonomic biases in the data. We intended the scope of the study to be global and cover different types of habitats and ecosystems (S1 Checklist); however, the majority of included studies were from North America (82%) and targeted mammals (90%), in particular large mammals (59% of the total number of effect sizes) (Fig 1). Furthermore, we were unable to evaluate whether variation in effect size was associated with different regions at smaller geographic scales because there was insufficient within-region replication to do so (e.g., different countries within Europe, or different provinces/states within Canada/USA). The taxonomic bias strongly limits the conclusions we can draw for taxa other than large mammals. However, we are less concerned about the geographic bias, because roads and traffic are essentially the same around the world.

### Effectiveness of fencing in reducing road-kill

Overall, we found that fences, with or without crossing structures, reduce road-kill by 54%. This finding supports the previous recommendation based on the opinion of a working group of seven US experts, that wildlife fencing, with or without wildlife crossing structures, is effective for reducing wildlife-vehicle collisions [9]. Interestingly, when analyzed separately, fencing alone reduced road-kill by 86% while crossing structures combined with fencing reduced road-kill by 51% (Fig 5). We suggest that this apparent reduction in the effectiveness of fencing when paired with crossing structures is due not to the addition of crossing structures *per se*, but rather to systematic differences among studies in fencing attributes or study design. For example, mitigation that combines crossing structures with fencing may tend to use shorter funnel or ‘wing’ fencing compared to fencing-only designs, which may fence longer stretches of roads. In our sample, in studies involving fencing alone, the average fence length was nearly triple that of studies of fencing combined with crossing structures [11406.8 m ± 4666.4 (1 SE) vs. 4041.5 m ± 855.4, respectively]. To adequately test this explanation, a comparison of studies involving fencing alone versus fencing combined with crossing structures, for the same fence length, would be required; however, there was not a large enough sample size to do so. Regarding study design, nearly half of the effect sizes from studies involving crossing structures and associated fencing employed a CI design, whereas none of the fencing-only studies did. This difference may have resulted in a stronger apparent effect of fencing alone, because BA and BACI studies have higher inferential strength than CI studies [20–23]. Overall, our results provide quantitative evidence that to reduce road-kill, mitigation should include wildlife fencing.

Unfortunately, we were unable to investigate the influence of various fence attributes such as fence height, mesh size, presence of dig barriers, overhangs or outriggers, fence-end treatments, or the level of fence maintenance, because there was not enough information reported within studies or variation within fence attributes to do so. Fence length is the only attribute we were able to evaluate and it was not associated with large mammal or amphibian and reptile fence effectiveness in reducing road-kill (Fig 4). In contrast, Huijser *et al*. [16] found that short fences (≤ 5 km road length) had lower and more variable effectiveness in reducing large mammal-vehicle collisions than long fences (> 5 km). In our data, we had a wide range in fence length for both fence types [large mammal fencing: 708−32200 (7357.95 ± 2121.9) m; amphibian and reptile fencing: 90−2000 (1333.24 ± 191.68) m], and an effect of fence length did not appear to be confounded by a variable that influenced mitigation effectiveness such as study design or road type. We note that our data, while not significant, did follow a similar trend as Huijser *et al*. [16], in that longer fences showed greater reductions in road-kill for large mammals than short fences. It is possible that a larger number of effect sizes is necessary to detect an association between fence length and average effect size. However still, our data do not provide sufficient evidence to draw conclusions on the association of fence length and effectiveness in reducing road-kill. Addressing this question will require more research and better data reporting.

The ‘fence-end issue’–in which road-kills are concentrated at the ends of wildlife fencing—may confound our understanding of the effectiveness of fences [17, 44–47]. If there is elevated road-kill immediately adjacent to fence-ends, the effectiveness of the fencing may be overestimated if road-kill in these locations is not included in the mortality estimates. Of the 25 studies that involved fencing (with or without crossing structures), only six (corresponding to 22 of 58 effect size estimates) measured road-kill beyond the fence-ends. Indeed, our results suggest that studies not accounting for fence-end issues may overestimate effectiveness: We observed larger average effect sizes for studies that did not measure road-kill beyond the fence-ends [1.14 (95% CI: 0.70, 1.58), *n* = 36; 59%] compared to studies that did [0.73 (95% CI: −0.03, 1.49), *n* = 22; 46%]. These results suggest that researchers should include an assessment of road-kill in areas immediately adjacent to fence-ends, and moreover, beyond the fence-ends when evaluating fence effectiveness. Authors should also clearly state the methods they used in their evaluation of effectiveness, reporting how far past fence-ends road-kill was sampled, the spatial accuracy of collision or carcass data, and provide road-kill data separately for areas immediately adjacent to fence-ends and beyond the fence-ends. Second, higher concentrations of road-kill at fence-ends should be considered indicative that fencing is not completely effective in reducing road-kill. By definition, road sections with relatively long and contiguous fencing (e.g., at least several kilometers) are less likely to have a fence-end issue than relatively short sections of fencing (e.g. up to several meters) [48]. Therefore, observations of elevated road-kill just past the ends of fencing is most likely because the fenced road sections are too short. Unless fencing is applied to the entire length of the roadway, the potential for the fence-end issue will always be present. If fencing the entire roadway is not possible, long(er) and contiguous fencing is needed to reduce or dilute the fence-end issue [16].

### Effectiveness of crossing structures in reducing road-kill

Crossing structures were not effective at reducing road-kill unless fences were present (Fig 5). One could argue that crossing structures without fencing are not intended to reduce-kill in any case, but rather to increase movement of animals across roads. However, the fact that some researchers measure road mortality before and after the installation of a crossing structure without fencing (e.g., [49]) means that reduced road-kill was at least part of the objective for the structure. Situations in which crossing structures have been installed without fencing include: (i) structures on small roads and railroads [50], (ii) small-animal passages in locations where snow clearing equipment would destroy small-animal fencing, (iii) situations where the movement paths of the target animals are known, (iv) situations where the crossing structure is intended for arboreal mammals (e.g., [51–53]), and (v) locations where large numbers of road-killed animals have been observed [49, 51]. Our results suggest that installing crossing structures alone is not effective for mitigating road-kill. Although we only had a small number of effect sizes for crossing structures alone (6 effect sizes from 3 studies), the data do not suggest that a larger sample size would produce a positive effect of crossing structures on mitigating road-kill because the mean effect size for crossing structures alone was slightly negative (Fig 5). If the goal of a crossing structure includes reducing road-kill, fences are required for effective mitigation.

### Expensive versus inexpensive mitigation measures for reducing road-kill

Our results suggest that expensive mitigation measures reduce large mammal road-kill much more than inexpensive measures. We observed an 83% reduction in road-kill for fencing with crossing structures, and a 57% reduction for animal detection systems, compared with only 1% for wildlife reflectors. While manufacturers often claim that reflectors are a scientifically proven method for reducing deer-vehicle collisions [54, 55], their long-term effectiveness is rarely considered and road planners should not take these claims at face value [13]. For example, while wildlife may initially respond to reflectors, this response generally declines over time as the animals habituate [56]. High-quality experiments testing effectiveness should be undertaken prior to widespread implementation (see Rytwinski *et al*. [22], van der Ree *et al*. [23] for standards for such studies). The cost-benefit of measures should also be considered because many of the more expensive measures (e.g. animal detection systems, crossing structures with associated fencing), have shown high returns on investment, with the ongoing benefits exceeding their costs over time [9, 57]. Overall road agencies should not assume (nor represent) they have mitigated large mammal road mortality through existing inexpensive measures such as wildlife reflectors; more expensive measures are required.

### Influence of study design

Studies that include ‘before data’ (BA and BACI studies) are much better able to detect effectiveness of mitigation measures on road-kill than those that do not (CI studies). This was expected based on considerations of relative inferential strength [20–23]. A particular problem with CI studies for evaluation of mitigation measures for road-kill is that selection of control sites is likely inadequate. It is quite common for mitigation measures to be implemented at high road-kill sites [58]. In CI studies, where we have only ‘after’ data (by definition), the measured road-kill at mitigation sites may be similar to control sites. However, this can mask an effect of mitigation, because the pre-existing road-kill at mitigation sites was likely higher than at control sites as usually high road-kill sites are mitigated and only low road-kill sites are left to be selected as control sites. Thus, in CI studies researchers are likely to incorrectly conclude that mitigation has little or no effect on reducing road-kill. If we had ‘before’ data for both the impact and control sites (i.e., a BACI design), we would know that the pre-existing road-kill at mitigation sites is higher than at control sites, and this difference would be incorporated in the analysis. We evaluated this possibility using ‘before’ data from the large mammal BACI studies in our sample (*n* = 20). As expected, we observed higher road-kill at impact sites (9.03 dead animals or wildlife-vehicle collisions/km/year ± 3.02 SE) compared to control sites (5.86 dead animals or wildlife-vehicle collisions/km/year ± 2.15 SE) before the mitigation was installed (two-tailed t-test: *t* = 2.94, df = 19, *p* = 0.009). This suggests that before data are necessary for evaluation of mitigation effectiveness for road mortality.

### Review limitations

There were not enough data to test many of the questions road planners have about the effectiveness of road-kill mitigation measures. For example, our sample of 99 was too small to answer: ‘Do other mitigation measures reduce road-kill (e.g., measures to reduce traffic volume and/or speed, temporary road closures, or increasing visibility through roadway lighting)?’, or ‘How do the attributes of crossing structures and fences influence their effectiveness (e.g., presence of dig barriers, overhangs or outriggers, fence-end treatments, mesh size, height, numbers and spacing of crossing structures and fenced sections etc.)?’ One of the main reasons for this small sample size was that many of the studies that we initially considered lacked suitable data for extraction, or the total sample size was too small to calculate an effect size. On the other hand, even if we were able to include these other studies, it is likely we would not have had a large enough sample size to address all the questions of interest for two reasons. First, there was often not enough variation in the values of the predictor variables to adequately test whether they influenced the effectiveness of road-kill mitigation measures. For example, there was little variation in fence height within a given type of fencing. Second, information about candidate predictor variables was often not reported [e.g., distance between crossing structures was reported in 32% of studies and length of crossing structures in 24% of studies]. To answer the remaining questions we need better research and data reporting for a broader range of mitigation measures.

### Towards better evaluations of mitigation effectiveness

To improve evaluations of mitigation effectiveness, we make the following recommendations for future studies. First, study designs should incorporate data collection before the mitigation is applied. A particular benefit of a BACI is that this design controls for any pre-existing biases or differences between control and impact sites within the analysis such as non-random assignment of impact and control sites. Second, we recommend *a minimum* study duration of four years for BA, and *a minimum* of either four years or four sites for BACI. We acknowledge that our recommendation to collect road-kill data before the mitigation is applied and/or to knowingly leave some sites unmitigated (i.e., control sites) may pose a risk to some wildlife populations. For example, where the target species is rare, threatened, or at risk of rapid local extinction due to road mortality, this recommendation may not always be acceptable. However, we argue that the long-term threat to wildlife populations by installing ineffective, untested mitigation is equally unacceptable. If trade-offs and compromises had to be made, we recommend to conduct one scientifically rigorous study that will contribute new knowledge on mitigation effectiveness rather than numerous poorly-designed studies [23]. Third, the above recommendations should be included in the early stages of a road project and road agency budgets should be adapted to these standards [20–23, 59]. Lastly, we recommend better coordination among road mitigation studies, since standardized methods and controlled protocols allow for much stronger meta-analyses and enhanced understanding of the effectiveness of mitigation measures. For example, the approach of coordinated distributed experiments (CDEs) can increase the quality of study designs and augment sample sizes (e.g., [60]) because road agencies can more easily pool money for research across a number of projects and plan more comprehensive monitoring programmes that include experimental study designs [22].

To better facilitate quantitative reviews, we have several recommendations for reporting of future studies. First, authors should provide raw data in an appendix or data archiving site. Road-kill data should be reported for each year before and after implementation/modification of the mitigation measure, and for each control and impact site *separately*. In other words, road-kill data should not be combined across years and/or sites and authors should clearly distinguish before, during, and after mitigation implementation/modification periods. Mortality data should also be recorded separately for each species or species group wherever possible. Second, authors should include: (i) test statistic(s) (e.g., *t*-values and *df* from a *t*-test comparing impact and control sites), and/or summary statistics (e.g. means *and* associated variances) from which an effect size can be calculated, and (ii) the sample sizes or the exact *p*-value if a test statistic was reported. Third, authors should include information on: (i) study locations such as vegetation cover types, proximity to human activities etc., (ii) road(s) and traffic such as road type, age of the road, number of lanes, traffic volume, and posted speed limit, (iii) the study design such as frequency of monitoring, method of monitoring, spatial accuracy of collision or carcass data, and whether and how far data were collected beyond fence-ends, (iv) attributes of each mitigation measure such as length, height, width, openness ratio of crossing structures, construction material, substrate material, age of measure relative to road age, distance to natural cover, number and spacing of measures (or provide a map and scale), whether other measures were incorporated, mesh size of fencing, whether there are top and/or bottom modifications to fencing, whether there are escapes provided in fencing etc., and (v) the overall project such as the level of mitigation maintenance, project costs etc. If this information is already available in another published study, authors should direct readers to that information. We had to exclude many studies from our analysis because they did not include the information outlined above. If we are to further our understanding of road-kill mitigation effectiveness, it is essential we make all monitoring data available and provide comprehensive information on study locations, study designs, road(s) and traffic, and the attributes of mitigation measures being evaluated.

## Conclusions and Implications

Our results highlight several key points of consideration for road planners and researchers when at least one of the goals of mitigation is to reduce road-kill. First, mitigation for road-kill should include wildlife fencing. Second, for large mammals, current animal detection systems can reduce road-kill, though not as effectively as wildlife fencing. Third, if the goal of a crossing structure includes reducing road-kill, fences must be included. Fourth, there is little or no evidence that other mitigation measures aimed at affecting driver or animal behaviour, including wildlife reflectors, reduce road-kill. We suggest that inexpensive measures such as reflectors should not be used until and unless their effectiveness is demonstrated using a high-quality experimental approach. Finally, proper evaluation and interpretation of the effectiveness of mitigation measures in reducing road-kill should include collection of road-kill data before mitigation measures are implemented, and should include *a minimum* study duration of four years for BA designs, and *a minimum* of either four years or four sites for BACI designs.

## Supporting Information

S1 Text Accounting for within-study nonindependence. (DOCX)

S1 Table Summary of research questions and predictor variables. Category or Range = levels of categorical variables, or descriptive statistics of continuous variables (SE = standard error of the mean); *n* = number of effect sizes in the analysis; Evaluation method = the method used to evaluate the fitted model, either information theoretic approach using AICc or null hypothesis testing using one-tailed *P*-values). Shaded questions are those that could not be addressed because of insufficient sample sizes; shaded predictor variables are those that could not be included because of insufficient variation.
(DOCX)

S2 Table Studies included in the meta-analysis and associated countries, mitigation categories, study designs, taxa, species or lowest level taxonomic groups, total study durations (years), whether or not data were collected during construction of the mitigation measure, road types, fence lengths (m), whether or not road-kill was monitored beyond the ends of the fencing, impact (*N*_T_ = impact sites or after monitoring period) and control group (*N*_C_ = control sites or before monitoring period) sample sizes, effect sizes (*d*), and variances for *d* (se). (DOCX)

S1 Fig PRISMA literature search flow diagram. (DOCX)

S2 Fig Relationship between effect sizes (*d*) and standard error to assess publication bias. Dashed line is the summary mean-weighted effect size from random-effect meta-analysis across 99 effect sizes from 50 studies.
(DOCX)

S3 Fig Scatterplot of effect size (*d*) versus total study duration (years) within the subset of BA+BACI study designs (*n* = 66). Symbol size is proportional to the weight (inverse of the sampling variance) of the effect size; smaller symbols correspond to effect sizes with lower weights.
(DOCX)

S1 Reference List Studies included in the meta-analysis. (DOCX)

S1 Checklist PRISMA Checklist. (DOCX)
