# Supplementary material for: How Effective Is Road Mitigation at Reducing Road-Kill? A Meta-Analysis
Source: PLoS One. 2016 Nov 21;11(11):e0166941. doi: 10.1371/journal.pone.0166941 (PMC5117745; doi:10.1371/journal.pone.0166941)
Supplement: S1 Text — (DOCX) [file pone.0166941.s001.docx]

**S1 Text**

*Accounting for within-study nonindependence*

**Methods**

We used two different but related datasets in our analyses. The complete dataset (*n* = 99) treated each effect size estimate as independent. By contrast, the synthetic effect size dataset (*n* = 67) was derived by pooling multiple effect sizes corresponding to different taxa from a single study. Many studies generated multiple effect size estimates, with different estimates corresponding to different species or taxa, different experiments (e.g., different impact versus control comparisons), and/or different mortality responses i.e., dead animal counts and wildlife-vehicle collisions.

Using multiple effect size estimates within a study introduces two problems: (1) the standard summary effect treats each effect size as an independent observation, thereby giving studies with multiple estimates more weight in the analysis; and (2) the analysis ignores the possibility of within-study correlations among effect size estimates, potentially leading to an overestimated precision of the summary effect [1, 2]. To address this potential bias, we calculated the intra-class correlation (ICC) based on the within- and between- study variances in effect size using Eq. 2 and 3 of Foy [3]. We then calculated a synthetic effect size for each “evaluation” (defined in the next paragraph), as well as a synthetic within-study variance based on the ICC Eq. 24.1 and Box 24.1(5) of Borenstein *et al*. [1]. To determine whether our results were biased, we then compared results obtained when treating each effect size as independent to the results obtained when using the synthetic effect size dataset.

To pool effect size estimates within a study, we first defined *a priori* what we considered to represent different “evaluations”. If within a study, multiple effect size estimates reflected different (a) study designs, (b) mitigation measures, (c) road types, (d) crossing structure types, (e) fencing types, (f) study locations (which implies different roads, or different crossing structures or fences), and/or (g) responses (i.e., dead animal counts vs. wildlife-vehicle collisions), they were treated as independent. On the other hand, multiple estimates reflecting different taxa were not treated as independent. Fifteen studies included multiple effect size estimates. For seven of these 15 studies, we pooled effect size estimates over taxa. There were two situations where taxonomic pooling resulted in > 2 effect size estimates due to differences in one or more variables (a) - (g) listed above e.g., half the estimates were in one study location and the other half were in a different study location [4, 5]. For the remaining eight studies, each effect size estimate was treated as independent since they reflected differences in (a) - (g).

**Results**

One-way analysis of variance, using all (*n* = 99) effect size estimates and m = 67 “evaluations”, yielded within (residual) and between-group estimates of 1.48 and 2.80 respectively, yielding an estimated intra-class correlation (see Shrout and Fleiss [6]) of ICC= 0.387, assumed independent of group (“evaluations”) and outcome. Results obtained by treating each effect size as an independent estimate, (complete dataset) versus pooling to obtain a synthetic effect size for each study (synthetic dataset), were qualitatively similar (Fig. A and Table A) insofar as (a) the estimated overall effect sizes were comparable; (b) factors associated with variation in effect size in the complete dataset were also associated with variation in the synthetic dataset, at least for factors with sufficient sample size to permit analysis in both cases.


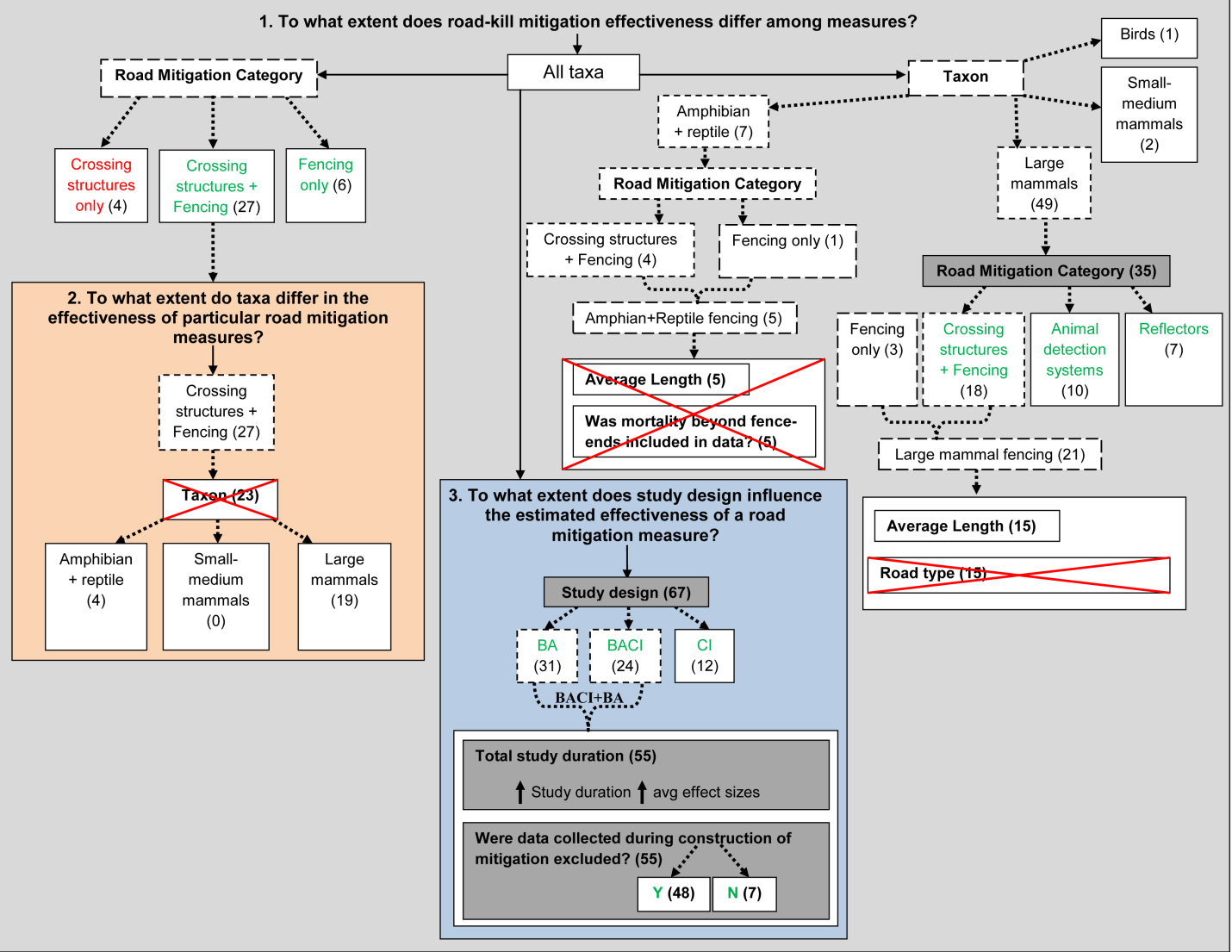


Fig A. Summary flow chart of the meta-analysis addressing our research questions using the synthetic effect size dataset (*n* = 67) and appropriate subsets (dashed boxes). Boxes enclosed by solid lines indicate predictor variables or subset categories under consideration. Shaded predictors were associated with road mitigation effectiveness. Subset categories in green indicate an overall average reduction in road-kill with road mitigation; red indicates an overall average increase in road-kill with road mitigation. Values in parentheses are the number of effect sizes. BA: Before-After; BACI: Before-After-Control-Impact; CI: Control-Impact study designs. Results using the synthetic effect size dataset did not qualitatively differ compared to using the complete dataset treating each effect size as an independent observation. A red ‘X’ identifies cases where sample sizes were too small to permit meaningful tests for effects using synthetic effect sizes.

Table A. Comparison of results for analyses based on the complete dataset versus the synthetic dataset. Shaded questions could not be addressed because of insufficient sample size. Predictor variables used in mixed-effects models are underlined. Potential associations with stricken out variables could not be investigated due to insufficient sample variation or sample size. *n* = number of effect sizes in the analysis; Null model = random-effects model; 95% CI = 95% confidence intervals; R^2^ = coefficient of determination; p = p-value; *Q_E_* = test statistic of residual heterogeneity; *Q_M_* = Omnibus test statistic of covariates; Mitigation Type: CF = crossing structures and associated fencing, C = crossing structures only, F = fencing only, ADS = animal detection systems, REFLECT = wildlife reflectors; Study design: BA = Before/After, BACI = Before-After-Control-Impact, and CI = Control/Impact study designs.

| Analysis | | Complete dataset | Synthetic effect size dataset |
| --- | --- | --- | --- |
| *Global* | |  |  |
|  | Overall average effect size | 0.75 (95% CI: 0.50, 1.00), *n*=99 | 0.84 (95% CI: 0.59, 1.08), *n*=67 |
| *Research questions* | |  |  |
| 1) | To what extent does road-kill mitigation effectiveness differ among measures? |  |  |
|  | (a) Overall, do crossing structures with associated fencing enhance the road-kill reduction effects of fencing *per se*? Prediction: Average effectiveness of CF will be greater than the average effectiveness of F. | CF: 0.95 (95% CI: 0.56, 1.35); F: 1.58 (95% CI: 0.42, 2.74); *Q_M_*=1.00, p=0.841 (one-tailed), R^2^=0.34, *n*=59 | CF: 1.40 (95% CI: 0.88, 1.92); F: 1.58 (95% CI: 0.42, 2.75); *Q_M_*=0.08, p=0.612 (one-tailed), R^2^=0.00, *n*=33 |
|  | (b) Overall, does fencing associated with crossing structures enhance the road-kill reduction effects of crossing structures *per se*? Prediction: Average effectiveness of CF will be greater than the average effectiveness of C. | CF: 0.95 (95% CI: 0.56, 1.35); C:-0.10 (95% CI:-1.11, 0.92); *Q_M_*=3.53, p=0.030 (one-tailed), R^2^=8.97, *n*=59 | CF: 1.42 (95% CI: 0.87, 1.96); C:-0.28 (95% CI:-1.55, 0.99); *Q_M_*=5.81, p=0.008 (one-tailed), R^2^=18.15, *n*=31 |
|  | (c) What mitigation measures are most effective for birds? |  |  |
|  | (d) What mitigation measures are most effective for large mammals? | Mitigation Category AICc (128.16) < Null model AICc (136.17), R^2^=52.81, *Q_E_*=61.18 (p=0.006), *Q_M_*=14.53 (p=0.001), *n*=39; CF: 1.83 (95% CI: 1.35, 2.31); ADS: 0.95 (95% CI: 0.37, 1.54); REFLECT: 0.14 (95% CI:-0.63, 0.91) | Mitigation Category AICc (111.23) < Null model AICc (123.55), R^2^=59.23, *Q_E_*=71.49 (p<0.0001), Q_M_=20.68 (p<0.0001), *n*=35; CF: 2.00 (95% CI: 1.51, 2.49); ADS: 0.98 (95% CI: 0.47, 1.49); REFLECT: 0.07 (95% CI:-0.64, 0.78) |
|  | (e) What mitigation measures are most effective for small-medium sized mammals? | ~~Mitigation category~~ |  |
|  | (f) What mitigation measures are most effective for amphibians and reptiles? | ~~Mitigation category~~ |  |
|  | (g) Which attributes (if any) of the most common measures are associated with effectiveness? |  |  |
|  | i. Which attributes of fencing are associated with effect size? |  |  |
|  | Within Large mammal fencing subset: | Road type AICc (68.19) < Null model AICc (69.17), R^2^ =26.00, *Q_E_*=31.43 (p=0.018), *Q_M_*=3.99 (P=0.046), *n*=19; ≥4- lane divided highways: 2.26 (95% CI: 1.58, 2.94); 1-2 lane roads: 1.03 (95% CI: -0.018, 2.07); Road length AICc (71.31) > Null model AICc (69.17) | ~~Road type~~; Road length AICc (57.02) > Null model AICc (53.89) |
|  | Within Small to medium sized mammal fencing subset: |  |  |
|  | Within Amphian+Reptile fencing subset: | Road length AICc (67.98) > Null model AICc (67.93); Was mortality beyond fence-ends included in data? AICc (71.31)> Null model AICc (67.93), *n*=14 | ~~Road length; Was mortality beyond fence-ends included in data?~~ |
|  | ii. Which attributes of crossing structures are associated with effect size? |  |  |
|  | Within Large mammal subset: |  |  |
|  | Within Small to medium sized mammal subset: |  |  |
|  | Within Amphian+Reptile subset: |  |  |
|  | Within Bird subset: |  |  |
| 2) | To what extent do taxa differ in the effectiveness of particular road mitigation measures? |  |  |
|  | Within Crossing structures and associated fencing subset: | Taxon AICc (185.84) < Null model AICc (188.20), R^2^=18.73, *Q_E_*=117.54 (p<0.0001); *Q_M_*=7.10 (p=0.029), *n*=47; Large mammals: 1.62 (95% CI: 57.47, 90.58); Amphibians+Reptiles: 0.53 (95% CI: -42.15, 50.49); Small-medium mammals: 0.30 (95% CI: 27.79, 113.974) | ~~Taxon~~ |
|  | Within Fencing only subset: |  |  |
|  | Within Crossing structures only subset: |  |  |
| 3) | To what extent does study design influence the estimated effectiveness of a road mitigation measure? | Study design AICc (346.22) < Null model AICc (352.77), R^2^=16.06, *Q_E_*=217.98 (p<0.0001), *Q_M_*=11.17 (p=0.004), *n*=99; BA: 1.09 (95% CI: 0.71, 1.47); BACI: 0.91 (95% CI: 0.43, 1.39); CI: 0.13 (95% CI: -0.31, 0.58) | Study design AICc (231.40) < Null model AICc (238.06), R^2^=12.32, *Q_E_*=309.01 (p<0.0001), *Q_M_*=7.08 (p=0.029), *n*=67; BA: 1.23(95% CI: 0.82, 1.65); BACI: 0.79 (95% CI: 0.29, 1.29); CI: 0.22 (95% CI: -0.40, 0.85) |
|  | Within BA+BACI design subset: |  |  |
|  | (b) Is there an association between effect size and total study duration? | Total study duration AICc (243.09) < Null model AICc (243.55), R^2^=9.65, *Q_E_*=176.80 (p<0.0001), *Q_M_*=2.68 (p=0.101), *n*=66 | Total study duration AICc (193.60) < Null model AICc (197.63), R^2^=12.71, *Q_E_*=252.74 (p<0.0001), *Q_M_*=4.55 (p=0.033), *n*=55 |
|  | (c) Is there an association between effect size and during construction data separation? | During construction data separation AICc (243.25) < Null model AICc (243.55), R^2^=9.63, *Q_E_*=177.73 (p<0.0001), *Q_M_*=2.53 (p=0.112), *n*=66; Yes: 0.95 (95% CI: 0.60, 1.30); No: 1.77 (95% CI: 0.80, 2.75) | During construction data separation AICc (195.09) < Null model AICc (197.63), R^2^=10.97, *Q_E_*=262.13 (p<0.0001), *Q_M_*=3.02 (p=0.082), *n*=55; Yes: 0.95 (95% CI: 0.60, 1.31); No: 1.87 (95% CI: 0.90, 2.85) |

**References**

1. Borenstein M, Hedge LV, Higgins JPT, Rothstein HR. Introduction to meta-analysis. West Sussex: John Wiley & Sons Ltd; 2009.

3. Foy P. Intraclass correlation and variance components as population attributes and measures of sampling efficiency in PIRLS. Hamburg, Germany: IEA Data Processing Center; 2001. Available: <http://www.iea.nl/fileadmin/user_upload/IRC/IRC_2004/Papers/IRC2004_Foy.pdf>.

2. Mengersen K, Jennions MD, Schmid CH. Statistical models for the meta-analysis of nonindependent data. In: Koricheva J, Gurevitch J, Mengersen K, editors. Handbook of meta-analysis in ecology and evolution. Princeton: Princeton University Press; 2013. pp. 255-283.

4. Mulder J. Reptielen en amfibieën als verkeerslachtoffer op wegen door en langs het Friese deel van het Fochteloërveen 1999 – 2009. WARF Bulletin. 2010; 13:12-25.

5. Niemi M, Jaaskelainen NC, Nummi P, Makela T, Norrdahl K. Dry paths effectively reduce road mortality of small and medium-sized terrestrial vertebrates. J Environ Manage. 2014; 144:51-57.

6. Shrout PE, Fleiss JL. Intraclass correlations: Uses in assessing rater reliability. Psychol Bull. 1979; 86: 420-428.
