# Supplementary material for: How Effective Is Road Mitigation at Reducing Road-Kill? A Meta-Analysis
Source: PLoS One. 2016 Nov 21;11(11):e0166941. doi: 10.1371/journal.pone.0166941 (PMC5117745; doi:10.1371/journal.pone.0166941)
Supplement: S1 Table — Category or Range = levels of categorical variables, or descriptive statistics of continuous variables (SE = standard error of the mean); n = number of effect sizes in the analysis; Evaluation method = the method used to evaluate the fitted model, either information theoretic approach using AICc or null hypothesis testing using one-tailed P-values). Shaded questions are those that could not be addressed because of insufficient sample sizes; shaded predictor variables are those that could not be included because of insufficient variation. (DOCX) [file pone.0166941.s002.docx]

S1 Table. Summary of research questions and predictor variables. Category or Range = levels of categorical variables, or descriptive statistics of continuous variables (SE= standard error of the mean); *n* = number of effect sizes in the analysis; Evaluation method= the method used to evaluate the fitted model, either information theoretic approach using AICc or null hypothesis testing using one-tailed *p*-values). Shaded questions could not be addressed because of insufficient sample sizes; shaded predictor variables could not be investigated because of insufficient sample variation.

| Research question | | Predictor variable | Category or Range (Mean ± SE) | *n* | Evaluation method |
| --- | --- | --- | --- | --- | --- |
| 1) | To what extent does road-kill mitigation effectiveness differ among measures? |  |  |  |  |
|  | (a) Overall, do crossing structures with associated fencing enhance the road-kill reduction effects of fencing *per se*? Prediction: Average effectiveness of CF will be greater than the average effectiveness of F. | Mitigation category | CF = Crossing structures with fencing; F= Fencing only | 59 | hypothesis testing |
|  | (b) Overall, does fencing associated with crossing structures enhance the road-kill reduction effects of crossing structures *per se*? Prediction: Average effectiveness of CF will be greater than the average effectiveness of C. | Mitigation category | CF = Crossing structures with fencing; C= Crossing structures only | 59 | hypothesis testing |
|  | (c) What mitigation measures are most effective for birds? |  |  |  |  |
|  | (d) What mitigation measures are most effective for large mammals? | Mitigation category | Crossing structures with fencing; animal detection systems; wildlife reflectors | 39 | information theoretic approach |
|  | (e) What mitigation measures are most effective for small-medium sized mammals? | Mitigation category |  |  |  |
|  | (f) What mitigation measures are most effective for amphibians and reptiles? | Mitigation category |  |  |  |
|  | (g) Which attributes (if any) of the most common measures are associated with effectiveness? |  |  |  |  |
|  | i. Which attributes of fencing are associated with effect size? |  |  |  |  |
|  | Within Large mammal fencing subset: |  |  |  |  |
|  |  | Length (log10) | 1150−32200 (7717.05 ± 2071.86) m | 19 | information theoretic approach |
|  |  | Road type | ≥4 lane divided highway/1-2 lane roads | 19 |  |
|  | Within Small to medium sized mammal fencing subset: |  |  |  |  |
|  | Within Amphian+Reptile fencing subset: |  |  |  |  |
|  |  | Length (log10) | 180−2000 (1339.64 ± 188.55) m | 14 | information theoretic approach |
|  |  | Was mortality beyond fence-ends included in data? | YES/NO | 14 |  |
|  | ii. Which attributes of crossing structures are associated with effect size? |  |  |  |  |
|  | Within Large mammal subset: |  |  |  |  |
|  | Within Small to medium sized mammal subset: |  |  |  |  |
|  | Within Amphian+Reptile subset: |  |  |  |  |
|  | Within Bird subset: |  |  |  |  |
| 2) | To what extent do taxa differ in the effectiveness of particular road mitigation measures? |  |  |  |  |
|  | Within Crossing structures and associated fencing subset: |  |  |  |  |
|  |  | Taxon | Amphibians+Reptiles; Small to medium sized mammals; Large mammals | 47 | information theoretic approach |
|  | Within Fencing only subset: |  |  |  |  |
|  | Within Crossing structures only subset: |  |  |  |  |
| 3) | To what extent does study design influence the estimated effectiveness of a road mitigation measure? | Study design | Before-After; Before-After-Control-Impact; Control-Impact | 99 | information theoretic approach |
|  | Within BA+BACI design subset: |  |  |  |  |
|  | (b) Is there an association between effect size and total study duration? | Total study duration | 2−22 (9.58 ± 0.61) years | 66 | information theoretic approach |
|  | (c) Is there an association between effect size and during construction data separation? | Were data collected during construction of mitigation excluded? | YES/NO | 66 | information theoretic approach |
