# Supplementary material for: How Effective Is Road Mitigation at Reducing Road-Kill? A Meta-Analysis
Source: PLoS One. 2016 Nov 21;11(11):e0166941. doi: 10.1371/journal.pone.0166941 (PMC5117745; doi:10.1371/journal.pone.0166941)
Supplement: S2 Table — (DOCX) [file pone.0166941.s003.docx]

S2 Table. Studies included in the meta-analysis and associated countries, mitigation categories, study designs, taxa, species or lowest level taxonomic groups, total study durations (years), whether or not data were collected during construction of the mitigation measure, road types, fence lengths (m), whether or not road-kill was monitored beyond the ends of the fencing, impact (*N*_T_ = impact sites or after monitoring period) and control group (*N*_C_= control sites or before monitoring period) sample sizes, effect sizes (*d*), and variances for *d* (se).

| Study* | Country | Mitigation category^a^ | Study design^b^ | Taxon^c^ | Taxon or Species | Total study duration | Were data collected during construction of mitigation? | Road type^d^ | Fence length | Was road-kill monitored beyond fence-ends? | N_T_ | N_C_ | *d* | se |
| --- | --- | --- | --- | --- | --- | --- | --- | --- | --- | --- | --- | --- | --- | --- |
| 1 | USA | CF | BACI | LM | *Odocoileus hemionus* | 8 | YES | ≥4 lane | 32200 | NO | 2 | 6 | 2.372 | 1.009 |
| 2 | USA | CF | BACI | LM | *Cervus elaphus nelsoni* | 9 | NO | ≥4 lane | 1150 | NO | 3 | 6 | 3.430 | 1.074 |
| 2 | USA | CF | BACI | LM | *Odocoileus* spp. | 9 | NO | ≥4 lane | 1150 | NO | 3 | 6 | 0.960 | 0.742 |
| 3 | Canada | CF | BACI | A | Anura | 3 | YES | 1-2 lane | 180 | YES | 4 | 10 | 7.088 | 1.403 |
| 3 | Canada | C | BACI | A | Anura | 3 | YES | 1-2 lane |  | N/A | 6 | 10 | -1.730 | 0.596 |
| 4 | USA | CF | BA | LM | *Cervus elaphus nelsoni* | 4 | NO | ≥4 lane | 1416 | YES | 2 | 2 | 1.045 | 1.066 |
| 5 | USA | CF | BA | LM | *Ovis canadensis nelson* | 4 | YES | ≥4 lane |  | UNKNOWN | 2 | 2 | 2.019 | 1.229 |
| 6 | USA | CF | BACI | LM | Artiodactyla | 8 | YES | ≥4 lane | 1930 | NO | 3 | 5 | 1.888 | 0.869 |
| 7 | USA | F | BACI | LM | *Odocoileus hemionus + Ovis canadensis* | 6 | YES | 1-2 lane | 14484 | NO | 1 | 5 | 2.028 | 1.242 |
| 8 | USA | CF | BA | LM | *Alce alces* | 11 | YES | ≥4 lane | 2816 | YES | 1 | 10 | 1.554 | 1.100 |
| 9 | USA | CF | BACI | LM | *Odocoileus virginianus clavium* | 8 | YES | 1-2 lane | 5200 | UNKNOWN | 1 | 3 | 1.421 | 1.259 |
| 10 | Portugal | CF | BACI | M | Mammalia | 2 | YES | ≥4 lane | 200 | UNKNOWN | 31 | 32 | 0.019 | 0.252 |
| 11 | USA | CF | BACI | LM | *Odocoileus hemionus* | 11 | NO | 1-2 lane | 4828 | NO | 1 | 10 | 0.739 | 1.061 |
| 12 | USA | CF | BACI | LM | *Odocoileus hemionus* | 5 | NO | various | 11430 | UNKNOWN | 2 | 2 | 0.522 | 1.017 |
| 13 | USA | CF | BACI | R | Testudines | 8 | YES | 1-2 lane | 225 | YES | 4 | 4 | -0.013 | 0.707 |
| 13 | USA | F | BACI | R | Testudines | 8 | YES | 1-2 lane | 550 | YES | 1 | 7 | 1.048 | 1.101 |
| 14 | Netherlands | CF | BA | LM | *Meles meles* | 20 | YES | various |  | UNKNOWN | 10 | 10 | -0.415 | 0.452 |
| 15 | Canada | CF | BA | LM | Mammalia (large) | 19 | NO | ≥4 lane | 10000 | YES | 15 | 4 | 4.293 | 0.895 |
| 15 | Canada | CF | BA | LM | Mammalia (large) | 19 | NO | ≥4 lane | 16000 | YES | 12 | 7 | 2.331 | 0.608 |
| 16 | USA | C | BA | M | Mammalia | 4 | YES | ≥4 lane |  | N/A | 2 | 2 | 0.341 | 1.007 |
| 17 | Canada | CF | CI | B | Aves | 4 | NA | 1-2 lane | 2000 | YES | 1 | 5 | -0.352 | 1.100 |
| 17 | Canada | CF | CI | SM | *Tamias striatus* | 4 | NA | 1-2 lane | 2000 | YES | 1 | 5 | -1.288 | 1.157 |
| 17 | Canada | CF | CI | SM | *Marmota monax* | 4 | NA | 1-2 lane | 2000 | YES | 1 | 5 | 0.358 | 1.100 |
| 17 | Canada | CF | CI | R | *Thamnophis sirtalis* | 4 | NA | 1-2 lane | 2000 | YES | 1 | 5 | 0.358 | 1.100 |
| 17 | Canada | CF | CI | B | *Anas platyrhynchos* | 4 | NA | 1-2 lane | 2000 | YES | 1 | 5 | 0.358 | 1.100 |
| 17 | Canada | CF | CI | SM | Muridae spp. | 4 | NA | 1-2 lane | 2000 | YES | 1 | 5 | 0.358 | 1.100 |
| 17 | Canada | CF | CI | SM | *Erethizon dorsatum* | 4 | NA | 1-2 lane | 2000 | YES | 1 | 5 | 0.576 | 1.108 |
| 17 | Canada | CF | CI | R | *Chrysemys picta* | 4 | NA | 1-2 lane | 2000 | YES | 1 | 5 | -3.522 | 1.495 |
| 17 | Canada | CF | CI | SM | *Procyon lotor* | 4 | NA | 1-2 lane | 2000 | YES | 1 | 5 | 0.666 | 1.112 |
| 17 | Canada | CF | CI | SM | *Tamiasciurus hudsonicus* | 4 | NA | 1-2 lane | 2000 | YES | 1 | 5 | 0.842 | 1.122 |
| 17 | Canada | CF | CI | SM | Leporidae spp. | 4 | NA | 1-2 lane | 2000 | YES | 1 | 5 | 0.358 | 1.100 |
| 17 | Canada | CF | CI | B | *Agelaius phoeniceus* | 4 | NA | 1-2 lane | 2000 | YES | 1 | 5 | 0.358 | 1.100 |
| 17 | Canada | CF | CI | SM | *Sciurus carolinensis* | 4 | NA | 1-2 lane | 2000 | YES | 1 | 5 | 0.306 | 1.099 |
| 17 | Canada | CF | CI | R | *Chelydra serpentina* | 4 | NA | 1-2 lane | 2000 | YES | 1 | 5 | -1.286 | 1.157 |
| 17 | Canada | CF | CI | LM | *Odocoileus virginianus* | 4 | NA | 1-2 lane | 2000 | YES | 1 | 5 | -0.214 | 1.097 |
| 18 | USA | CF | CI | M | Mammalia | 1 | NA | ≥4 lane | 2000 | NO | 3 | 4 | 0.419 | 0.772 |
| 19 | Finland | C | CI | SM | Mammalia | 1 | NA | 1-2 lane |  | N/A | 10 | 10 | 0.633 | 0.458 |
| 19 | Finland | C | CI | A | Amphibia | 1 | NA | 1-2 lane |  | N/A | 10 | 10 | 1.071 | 0.478 |
| 19 | Finland | C | CI | SM | Mammalia | 1 | NA | 1-2 lane |  | N/A | 10 | 10 | -0.551 | 0.456 |
| 19 | Finland | C | CI | A | Amphibia | 1 | NA | 1-2 lane |  | N/A | 10 | 10 | -0.310 | 0.450 |
| 20 | USA | CF | CI | A+R | Amphibia + Reptilia | 2 | NA | 1-2 lane | 1700 | NO | 1 | 3 | -1.767 | 1.313 |
| 20 | USA | CF | CI | LM | *Ursus americanus floridanus* | 2 | NA | 1-2 lane | 1700 | NO | 1 | 3 | 0.655 | 1.178 |
| 20 | USA | CF | CI | LM | *Odocoileus virginianus* | 2 | NA | 1-2 lane | 1700 | NO | 1 | 3 | -0.019 | 1.155 |
| 20 | USA | CF | CI | LM | *Canis latrans* | 2 | NA | 1-2 lane | 1700 | NO | 1 | 3 | 0.578 | 1.173 |
| 20 | USA | CF | CI | SM | *Urocyon cineroargenteus floridanus* | 2 | NA | 1-2 lane | 1700 | NO | 1 | 3 | 0.445 | 1.165 |
| 20 | USA | CF | CI | B | Aves | 2 | NA | 1-2 lane | 1700 | NO | 1 | 3 | 0.480 | 1.167 |
| 21 | USA | F | BA | B | *Sterna maxima* | 11 | YES | 1-2 lane |  | N/A | 6 | 5 | 1.259 | 0.662 |
| 22 | USA | CF | BACI | LM | Mammalia (large) | 7 | YES | 1-2 lane | 2220 | UNKNOWN | 3 | 4 | 1.696 | 0.890 |
| 23 | Canada | CF | BA | LM | Mammalia | 12 | YES | ≥4 lane |  | UNKNOWN | 6 | 6 | 2.063 | 0.715 |
| 24 | Finland | F | BACI | LM | Artiodactyla | 13 | YES | ≥4 lane | 26000 | NO | 10 | 3 | 2.826 | 0.861 |
| 25 | USA | ADS | BA | LM | Mammalia (large) | 10 | YES | 1-2 lane |  | N/A | 2 | 8 | 0.041 | 0.791 |
| 26 | USA | ADS | BACI | LM | Mammalia (large) | 11 | YES | 1-2 lane |  | N/A | 2 | 9 | -0.207 | 0.783 |
| 27 | USA | ADS | BACI | LM | Mammalia (large) | 10 | NO | 1-2 lane |  | N/A | 1 | 9 | 0.514 | 1.060 |
| 28 | USA | REFLECT | BACI | LM | *Odocoileus virginianus* | 7 | YES | various |  | N/A | 2 | 5 | -0.096 | 0.837 |
| 29 | USA | REFLECT | CI | LM | *Odocoileus hemionus* | 3 | NA | various |  | N/A | 3 | 3 | -0.823 | 0.850 |
| 30 | USA | REFLECT | CI | LM | *Odocoileus hemionus* | 3 | NA | 1-2 lane |  | N/A | 3 | 3 | -1.300 | 0.899 |
| 31 | USA | REFLECT | CI | LM | *Odocoileus virginianus* | 1 | NA | various |  | N/A | 10 | 10 | 0.033 | 0.447 |
| 32 | Canada | REFLECT | BACI | LM | *Odocoileus* spp. | 15 | YES | various |  | N/A | 2 | 4 | -0.577 | 0.882 |
| 33 | Canada | O | BACI | LM | *Odocoileus virginianus + Odocoileus hemionus* | 4 | YES | various |  | N/A | 1 | 3 | 3.671 | 1.738 |
| 34 | USA | O | BACI | R | Testudines | 3 | YES | various |  | N/A | 5 | 4 | -0.443 | 0.679 |
| 35 | USA | O | BACI | LM | *Odocoileus hemionus* | 5 | YES | various |  | N/A | 5 | 5 | 0.803 | 0.657 |
| 36 | USA | O | CI | LM | *Odocoileus hemionus* | 4 | NA | ≥4 lane |  | N/A | 4 | 4 | 0.182 | 0.709 |
| 37 | Canada | O | BACI | LM | *Cervus elaphus* | 16 | YES | 1-2 lane |  | N/A | 8 | 8 | 0.885 | 0.524 |
| 37 | Canada | O | BACI | LM | *Ovis canadensis* | 16 | YES | 1-2 lane |  | N/A | 8 | 8 | -0.187 | 0.501 |
| 38 | Australia | O | CI | SM | *Phascolarctos cinereus* | 5 | NA | various |  | N/A | 5 | 4 | 0.917 | 0.705 |
| 39 | Canada | CF | BA | LM | Mammalia (large) | 21 | YES | ≥4 lane | 1700 | NO | 16 | 5 | 5.429 | 0.982 |
| 39 | Canada | CF | BA | LM | Mammalia (large) | 22 | YES | ≥4 lane | 6400 | NO | 5 | 17 | 1.105 | 0.535 |
| 39 | Canada | CF | BA | LM | Mammalia (large) | 21 | YES | ≥4 lane | 14030 | NO | 2 | 19 | 1.045 | 0.761 |
| 40 | USA | O | CI | LM | *Odocoileus* spp. | 2 | NA | 1-2 lane |  | N/A | 3 | 3 | 0.681 | 0.840 |
| 41 | USA | REFLECT | CI | LM | *Odocoileus virginianus + Odocoileus hemionus* | 4 | NA | 1-2 lane |  | N/A | 4 | 4 | 3.186 | 1.066 |
| 42 | Netherlands | CF | BA | R | *Zootoca vivipara* | 10 | YES | various | 1950 | NO | 6 | 4 | 0.721 | 0.665 |
| 42 | Netherlands | CF | BA | R | *Coronella austriaca* | 10 | YES | various | 1950 | NO | 6 | 4 | -0.778 | 0.669 |
| 42 | Netherlands | CF | BA | R | *Natrix natrix* | 10 | YES | various | 1950 | NO | 6 | 4 | 0.497 | 0.655 |
| 42 | Netherlands | CF | BA | R | *Vipera berus* | 10 | YES | various | 1950 | NO | 6 | 4 | 0.654 | 0.662 |
| 42 | Netherlands | CF | BA | R | *Zootoca vivipara* | 10 | YES | various | 1000 | NO | 8 | 2 | 0.452 | 0.797 |
| 42 | Netherlands | CF | BA | R | *Coronella austriaca* | 10 | YES | various | 1000 | NO | 8 | 2 | -0.700 | 0.806 |
| 42 | Netherlands | CF | BA | R | *Natrix natrix* | 10 | YES | various | 1000 | NO | 8 | 2 | 2.292 | 0.942 |
| 42 | Netherlands | CF | BA | R | *Vipera berus* | 10 | YES | various | 1000 | NO | 8 | 2 | 4.629 | 1.303 |
| 43 | USA | O | BA | LM | *Odocoileus virginianus* | 4 | YES | various |  | N/A | 3 | 1 | 1.137 | 1.223 |
| 43 | USA | O | BA | LM | *Odocoileus virginianus* | 7 | YES | various |  | N/A | 6 | 1 | 2.912 | 1.331 |
| 44 | USA | O | BA | LM | *Odocoileus virginianus* | 6 | YES | various |  | N/A | 3 | 3 | -0.938 | 0.860 |
| 45 | USA | O | BA | LM | *Odocoileus virginianus* | 4 | YES | various |  | N/A | 2 | 2 | 0.515 | 1.016 |
| 46 | Germany | O | BA | LM | *Cervus elaphus* | 6 | YES | 1-2 lane |  | N/A | 1 | 5 | -1.431 | 1.171 |
| 46 | Germany | O | BA | LM | *Capreolus capreolus* | 6 | YES | 1-2 lane |  | N/A | 1 | 5 | -0.985 | 1.132 |
| 46 | Germany | O | BA | LM | *Sus scrofa* | 6 | YES | 1-2 lane |  | N/A | 1 | 5 | -1.306 | 1.159 |
| 46 | Germany | O | BA | SM | Mammalia | 6 | YES | 1-2 lane |  | N/A | 1 | 5 | 0.876 | 1.124 |
| 47 | Germany | F | BA | SM | *Erinaceus europaeus* | 9 | YES | 1-2 lane | 2000 | NO | 4 | 5 | 1.142 | 0.723 |
| 48 | Canada | CF | BA | LM | *Alces alces* | 5 | YES | various | 23000 | NO | 3 | 2 | 1.908 | 1.094 |
| 48 | Canada | CF | BA | LM | *Alces alces* | 5 | YES | various | 12000 | NO | 2 | 3 | 3.240 | 1.372 |
| 48 | Canada | F | BA | LM | *Alces alces* | 5 | YES | various | 14000 | NO | 1 | 4 | 1.248 | 1.186 |
| 49 | USA | O | BACI | LM | *Odocoileus virginianus* | 8 | YES | various |  | N/A | 2 | 6 | 1.410 | 0.889 |
| 49 | USA | REFLECT | BACI | LM | *Odocoileus virginianus* | 8 | YES | various |  | N/A | 2 | 6 | 2.060 | 0.965 |
| 50 | Switzerland | ADS | BA | LM | Artiodactyla | 17 | YES | various |  | N/A | 10 | 7 | 1.222 | 0.536 |
| 50 | Switzerland | ADS | BA | LM | Artiodactyla | 17 | YES | various |  | N/A | 6 | 11 | 0.640 | 0.519 |
| 50 | Switzerland | ADS | BA | LM | Artiodactyla | 8 | YES | various |  | N/A | 5 | 3 | 0.714 | 0.747 |
| 50 | Switzerland | ADS | BA | LM | Artiodactyla | 9 | YES | various |  | N/A | 5 | 4 | 1.033 | 0.714 |
| 50 | Switzerland | ADS | BA | LM | Artiodactyla | 14 | YES | various |  | N/A | 6 | 8 | 2.086 | 0.669 |
| 50 | Switzerland | ADS | BA | LM | Artiodactyla | 14 | YES | various |  | N/A | 6 | 8 | 1.505 | 0.610 |
| 50 | Switzerland | ADS | BA | LM | Artiodactyla | 15 | YES | various |  | N/A | 7 | 8 | 1.360 | 0.574 |

*Studies cited in S1 Reference List.

^a^Mitigation Type: CF = crossing structures and associated fencing, F = fencing only, C = crossing structures only, ADS = animal detection systems, REFLECT = wildlife reflectors, and O = other mitigation.

^b^Study design: BA = Before/After, BACI = Before-After-Control-Impact, and CI = Control/Impact study designs.

^c^Taxon: LG= large mammals (≥ 10 kg), SM=small to medium sized mammals (< 10 kg), M = mammal (different sized or unspecified mammals), B = bird, R = reptile, and A = amphibian.

^d^Road type: ≥4 lane= ≥4 lane divided highways, 1-2 lane= 1-2 lane roads, various = various road types included.
