# Supplementary figures and images for: How Effective Is Road Mitigation at Reducing Road-Kill? A Meta-Analysis
Source: PLoS One. 2016 Nov 21;11(11):e0166941. doi: 10.1371/journal.pone.0166941 (PMC5117745; doi:10.1371/journal.pone.0166941)

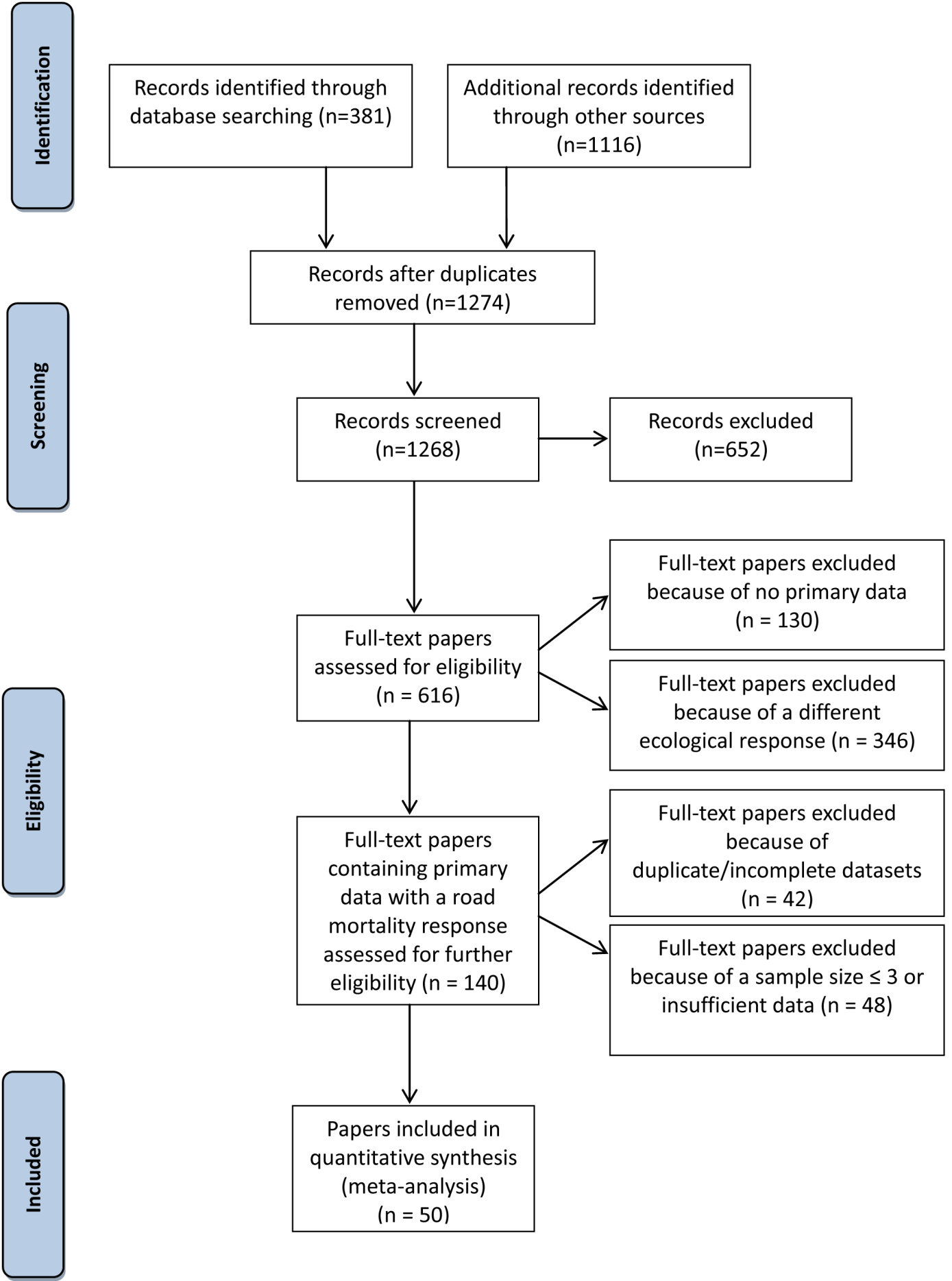


S1 Fig. PRISMA literature search flow diagram.

Supplement: S1 Fig — (DOCX) [file pone.0166941.s004.docx]
