# Supplementary material for: How Effective Is Road Mitigation at Reducing Road-Kill? A Meta-Analysis
Source: PLoS One. 2016 Nov 21;11(11):e0166941. doi: 10.1371/journal.pone.0166941 (PMC5117745; doi:10.1371/journal.pone.0166941)
Supplement: S2 Fig — Dashed line is the summary mean-weighted effect size from random-effect meta-analysis across 99 effect sizes from 50 studies. (DOCX) [file pone.0166941.s005.docx]

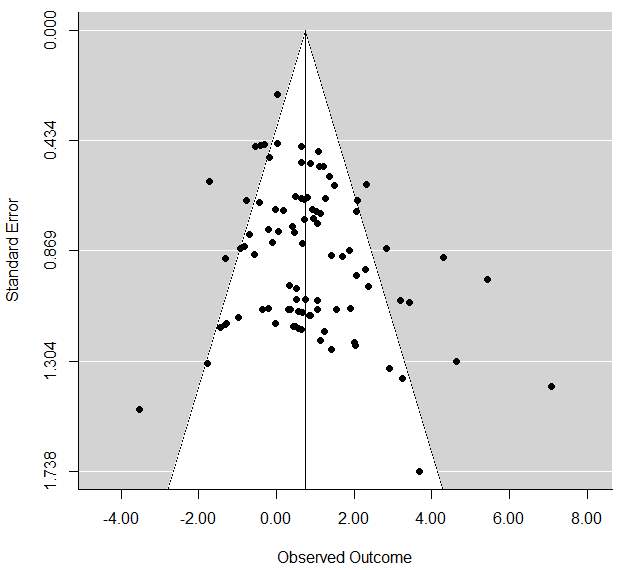


S2 Fig. Relationship between effect sizes (*d*) and standard error to assess publication bias. Dashed line is the summary mean-weighted effect size from random-effect meta-analysis across 99 effect sizes from 50 studies.
