# Supplementary material for: How Effective Is Road Mitigation at Reducing Road-Kill? A Meta-Analysis
Source: PLoS One. 2016 Nov 21;11(11):e0166941. doi: 10.1371/journal.pone.0166941 (PMC5117745; doi:10.1371/journal.pone.0166941)
Supplement: S3 Fig — Symbol size is proportional to the weight (inverse of the sampling variance) of the effect size; smaller symbols correspond to effect sizes with lower weights. (DOCX) [file pone.0166941.s006.docx]

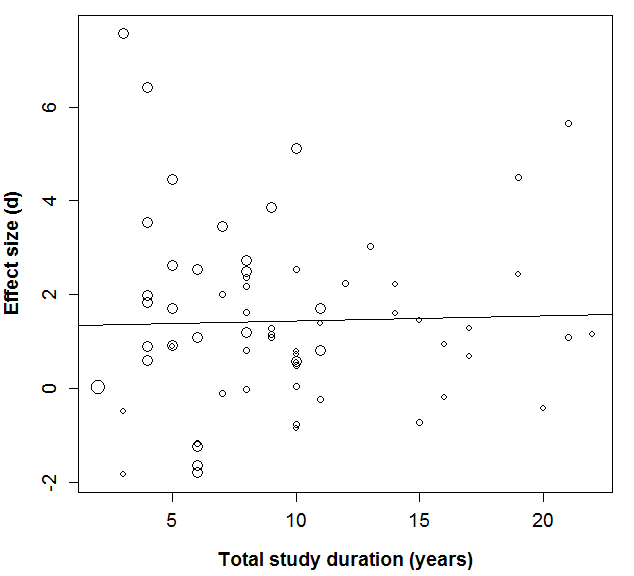


S3 Fig. Scatterplot of effect size (*d*) versus total study duration (years) within the subset of BA+BACI study designs (*n* = 66). Symbol size is proportional to the inverse variance weight of each effect size; smaller symbols correspond to effect sizes with lower weights.
