# Supplementary material for: How Effective Is Road Mitigation at Reducing Road-Kill? A Meta-Analysis
Source: PLoS One. 2016 Nov 21;11(11):e0166941. doi: 10.1371/journal.pone.0166941 (PMC5117745; doi:10.1371/journal.pone.0166941)
Supplement: S1 Reference List — (DOCX) [file pone.0166941.s007.docx]

S1 Reference List. Studies included in the meta-analysis.

1. Bissonette JA, Rosa S. An evaluation of a mitigation strategy for deer-vehicle collisions. Wildlife Biol. 2012; 18:414-423.
2. Gagnon JW. Evaluation of Desert Bighorn Sheep Overpasses along U.S. Highway 93 in Arizona. In: Proceedings of the 2013 International Conference on Ecology and Transportation; 2013. Available: <http://www.icoet.net/ICOET_2013/proceedings.asp>.
3. Cunnington G, Garrah E, Eberhardt E, Fahrig L. Culverts alone do not reduce road mortality in anurans. Ecoscience. 2014; 21: 69-78.
4. Dodd NL, Gagnon J, Boe S, Manzo A, Schweinsburg RE. Evaluation of measures to minimize wildlife-vehicle collisions and maintain permeability across highways: Arizona Route 260. Prepared for Arizona Department of Transportation, Phoenix, Arizona, USA; 2007-2012.
5. Gagnon JW. Evaluation of Desert Bighorn Sheep Overpasses along U.S. Highway 93 in Arizona. In: Proceedings of the 2013 International Conference on Ecology and Transportation; 2013. Available: <http://www.icoet.net/ICOET_2013/proceedings.asp>.
6. Craighead L, Craighead A, Oechsli L, Kociolek A. Bozeman pass post-fencing wildlife monitoring. Final Report. Prepared for Montana Department of Transportation, Helena, MT, USA; 2010.
7. McAllister K. A Wildlife Barrier Fence North of Wenatchee, Washington: Learning Experiences involving Rugged Country and Custom-Designed Wildlife Guards and Jumpouts. In: Proceedings of the 2013 International Conference on Ecology and Transportation; 2013. Available: <http://www.icoet.net/ICOET_2013/proceedings.asp>.
8. McDonald M. Glenn Highway Moose monitoring study. Final report prepared for Alaska Department of Transportation and Public Facilities, Anchorage, AK, USA; 1991.
9. Parker ID, Braden AW, Lopez RR, Silvy NJ, Davis DS, Owen CB. Effects of US 1 Project on Florida Key Deer Mortality. J Wildl Manage. 2008; 72:354-359.
10. Villalva P, Reto D, Santos-Reis M, Revilla E, Grilo C. Do dry ledges reduce the barrier effect of roads? Ecol Eng. 2013: 57:143-148.
11. Attah I. An evaluation of the effectiveness of wildlife crossings on Mule Deer and other wildlife. Dissertation, University of Nevada, Reno. 2012.
12. Lehnert ME, Bissonette JA. Effectiveness of highway crosswalk structures at reducing deer-vehicle collisions. Wildlife Soc B. 1997; 25:809-818.
13. Langen TA. Monitoring Functionality and Durability of the New York State Highway 30 Turtle Barrier and Adjacent Nesting Substrate. Final Report prepared for New York State Department of Transportation, Albany, NY, USA; 2012.
14. Dekker JJA, Bekker HGJ. Badger (*Meles meles*) road mortality in the Netherlands: the characteristics of victims and the effects of mitigation measures. Lutra. 2010; 53:81 - 92.
15. Clevenger AP, Chruszcz B, Gunson K. Highway Mitigation Fencing Reduces Wildlife-Vehicle Collisions. Wildlife Soc B. 2001; 29:646-653.
16. Elliott D. Effects of a purpose-built faunal underpass on activity and traffic-related mortality of wildlife in southern California. Dissertation, California State University, Fullerton, CA, USA. 2008.
17. Dillion Consulting Ltd. Terry Fox Drive Extension Project: Wildlife Guide System Monitoring Report, Year 3 of 3; and, Three Year Summary. Prepared for the City of Ottawa, Ottawa, ON, Canada; 2014.
18. McCollister MF, van Manen FT. Effectiveness of Wildlife Underpasses and Fencing to Reduce Wildlife–Vehicle Collisions. Journal of Wildlife Management. 2010; 74:1722-1731.
19. Niemi M, Jaaskelainen NC, Nummi P, Makela T, Norrdahl K. Dry paths effectively reduce road mortality of small and medium-sized terrestrial vertebrates. J Environ Manage. 2014; 144:51-57.
20. Walker G, Baber J. Wildlife use and interactions with structures constructed to minimize vehicle collisions and animal mortality along State Road 46, Lake County Florida. Prepared for Florida Department of Transportation, Tallahassee, Florida, USA; 2003.
21. Bard AM, Smith HT, Egensteiner ED, Mulholland R, Harber TV, Heath GW, et al. A simple structural method to reduce road-kills of royal terns at bridge sites. Wildlife Soc B. 2002; 30:603–605.
22. Huijser MP, Fairbank E, Camel-Means W. US 93 North Post-Construction Wildlife-Vehicle Collision and Wildlife Crossing Monitoring and Research on the Flathead Indian Reservation between Evaro and Polson, Montana Annual Report 2014. Prepared for Montana Department of Transportation, Helena, Montana; 2015.
23. Lee T. Trans-Canada Highway and Dead Man's Flat Underpass: Is highway mitigation cost-effective? In: Proceedings of the 2013 International Conference on Ecology and Transportation; 2013. Available: <http://www.icoet.net/ICOET_2013/proceedings.asp>.
24. Niemi M, Martin A, Tanskanen A, Numm P. How effective are wildlife fences in preventing collisions with wild ungulates? In: Richter V, Puky M, Seiler A, editors, Improving connections in a changing environment. Collection of short papers from the 2010 IENE Conference*.* (Varangy Akciocsoport Egyesulet - MTA Okologiai es Botanikai Kutatointezete - SCOPE Ltd., Budapest – Vacratot; 2010.
25. Dai Q, Young R, Vander Giessen S. Evaluation of an active wildlife-sensing and driver warning system at Trapper’s Point. FHWA-WY-09/03F, Department of Civil and Architectural Engineering, University of Wyoming, Laramie, Wyoming, USA; 2009.
26. Huijser M, Haas C, Crooks K. The reliability and effectiveness of an electromagnetic animal detection and driver warning system. Prepared for Colorado Department of Transportation – Research, Denver, Colorado, USA; 2012.
27. Huijser M, Holland TD, Kociolek A, Barkdoll AM, Schwalm JD. Animal-vehicle crash mitigation using advanced technology. Phase II: system effectiveness and system acceptance. Final Report prepared for Oregon Department of Transportation Research Unit, Salem, OR and laska Department of Transportation and Public Facilities and the Departments of Transportation in California, Indiana, Iowa, Kansas, Maryland, Montana, Nevada, New Hampshire, New York State, North Dakota, Pennsylvania, Wisconsin, and Wyoming, and Federal Highway Administration, Washington, DC, USA; 2009. Available: <http://www.oregon.gov/ODOT/TD/TP_RES/docs/Reports/2009/Animal_Vehicle_Ph2.pdf>.
28. Christensen K. Evaluation of Strieter‐Lite wild Animal Highway Warning Reflector System™ on reducing vehicle‐animal collisions. Montana Department of Transportation Research Programs; 2011-2013.
29. Reeve AF, Anderson SH. Ineffectiveness of Swareflex reflectors at reducing deer-vehicle collisions. Wildlife Soc B. 1993; 21:127–132.
30. Ford S, Villa S. Reflector use and the effect they have on the number of Mule Deer killed on California highways. Prepared for California Department of Transportation, Sacramento, California, USA; 1993.
31. Cottrell BH. Evaluation of deer warning reflectors in Virginia. Virginia Transportation Research Council, Charlottesville, Virginia, USA; 2003.
32. Sielecki LE. Evaluating the effectiveness of wildlife accident mitigation installations with the Wildlife Accident Reporting System (WARS) in British Columbia. In: Irwin CL, Garret P, McDermott KP, editors, Proceedings of the International Conference on Ecology and Transportation. North Carolina State University, Raleigh, USA; 2001.
33. Found R, Boyce MS. Warning signs mitigate deer-vehicle collisions in an urban area. Wildlife Soc B. 2011; 35:291-295.
34. Johnson G. Testing the effectiveness of turtle crossing signs as a conservation measure. Final Report prepared for St. Lawrence River Research and Educational Fund, New York Power Authority, New York, USA; 2011.
35. Sullivan TL, Williams AF, Messmer TA, Hellinga LA, Kyrychenko SY. Effectiveness of temporary warning signs in reducing deer-vehicle collisions during mule deer migrations. Wildlife Soc B. 2004; 32:907–915.
36. Reed DF, Woodard TN. Effectiveness of highway lighting in reducing deer-vehicle accidents. J Wildl Manage. 1981; 45:721-726.
37. Bertwistle J. The effects of reduced speed zones on reducing Bighorn Sheep and Elk collisions with vehicles on the Yellowhead Highway in Jasper National Park. In: Evink G, Garret P, Zelgler D, editors, Proceedings of the Third International Conference on Wildlife Ecology and Transportation, pp 101-109. Missoula, Montana; 1999.
38. Dique DS, Thompson J, Preece HJ, Penfold GC, de Villiers DL, Leslie RS. Koala mortality on roads in south-east Queensland: the koala speed-zone trial. Wildlife Research. 2003; 30:419-426.
39. Clevenger AP, Barrueto M. Trans‐Canada Highway Wildlife Monitoring and Research Final Report 2014. Part B: Research. Prepared for Parks Canada Agency Radium Hot Springs, British Columbia; 2014.
40. Wood P, Wolfe ML. Intercept Feeding as a Means of Reducing Deer-Vehicle Collisions. Wildlife Society Bulletin. 1988; 16:376-380.
41. Schafer JA, Penland S, Carr WP. Effectiveness of wildlife warning reflectors in reducing deer-vehicle accidents in Washington state. Prepared for Washington State Department of Transportation, Olympia, WA, USA; 1984.
42. Mulder J. Reptielen en amfibieën als verkeerslachtoffer op wegen door en langs het Friese deel van het Fochteloërveen 1999 – 2009. WARF Bulletin. 2010; 13:12-25.
43. DeNicola AJ, Williams SC. Sharpshooting suburban white-tailed deer reduces deer–vehicle collisions. Human–Wildlife Conflicts. 2008; *2*:28–33.
44. Doerr ML, McAninch JB, Wiggers EP. Comparison of 4 Methods to Reduce White-Tailed Deer Abundance in an Urban Community. Wildlife Soc B. 2001; 29:1105-1111.
45. Engeman RM, Guerrant T, Dunn G, Beckerman SF, Anchor C. Benefits to rare plants and highway safety from annual population reductions of a “native invader,”
    white-tailed deer, in a Chicago-area woodland. Environ Sci Pollut Res. 2014; 21:1592–1597.
46. Lutz VWB. Ergebnisse der Anwendung eines sogenannten Duftzaunes zur
    Vermeidung von Wildverlusten durch den Straßenverkehr nach
    Gehege- und Freilandorientierungen. Z Jagdwiss. 1994; 40:91-108.
47. Reichholf J. Üeber die Wirkung von Igelschutzzäeunen im Siedlungsrandbereich. Säeugetierkundliche Mitteilungen. 1984; 31:267.
48. Bouffard M, Leblanc Y, Bedard Y, Martel D. Impacts de clotures métalliques et de passages fauniques sur la sécurité routière et le déplacement des orignaux le long de la route 175 au Quebec. Le Naturaliste Canadien. 2012; 136:8-15.
49. Rogers EI. An Ecological Landscape Study of Deer-vehicle Collisions in Kent County, Michigan. Report for the Michigan State Police, Office of Highway Safety and Planning. White Water Associates, Inc., Amasa, Michigan, USA; 2004.
50. Romer J, Mosler-Berger C. Preventing vehicle-wildlife accidents - The animal detection system CALSTROM. In: Proceedings of the 2003 Infra Eco Network Europe Conference: Habitat Fragmentation Due to Transport Infrastructure and Presentation of the COST 341 Action, Brussels, Belgium; 2003. Available: http://www.iene.info/.
